# 14-3-3γ Prevents Centrosome Amplification and Neoplastic Progression

**DOI:** 10.1038/srep26580

**Published:** 2016-06-02

**Authors:** Amitabha Mukhopadhyay, Lalit Sehgal, Arunabha Bose, Anushree Gulvady, Parijat Senapati, Rahul Thorat, Srikanta Basu, Khyati Bhatt, Amol S. Hosing, Renu Balyan, Lalit Borde, Tapas K. Kundu, Sorab N. Dalal

**Affiliations:** 1 Advanced Centre for Treatment Research and Education in Cancer, Tata Memorial Center, Mumbai 410210, India; 2 Transcription and Disease Laboratory, Molecular Biology and Genetics Unit, Jawaharlal Nehru Centre for Advanced Scientific Research, Bangalore 560064, India; 3 National Institute of Immunology, New Delhi 110067, India; 4 Department of Biological Sciences, Tata Institute of Fundamental Research, Mumbai 400005, India

## Abstract

More than 80% of malignant tumors show centrosome amplification and clustering. Centrosome amplification results from aberrations in the centrosome duplication cycle, which is strictly coordinated with DNA-replication-cycle. However, the relationship between cell-cycle regulators and centrosome duplicating factors is not well understood. This report demonstrates that 14-3-3γ localizes to the centrosome and 14-3-3γ loss leads to centrosome amplification. Loss of 14-3-3γ results in the phosphorylation of NPM1 at Thr-199, causing early centriole disjunction and centrosome hyper-duplication. The centrosome amplification led to aneuploidy and increased tumor formation in mice. Importantly, an increase in passage of the 14-3-3γ-knockdown cells led to an increase in the number of cells containing clustered centrosomes leading to the generation of pseudo-bipolar spindles. The increase in pseudo-bipolar spindles was reversed and an increase in the number of multi-polar spindles was observed upon expression of a constitutively active 14-3-3-binding-defective-mutant of cdc25C (S216A) in the 14-3-3γ knockdown cells. The increase in multi-polar spindle formation was associated with decreased cell viability and a decrease in tumor growth. Our findings uncover the molecular basis of regulation of centrosome duplication by 14-3-3γ and inhibition of tumor growth by premature activation of the mitotic program and the disruption of centrosome clustering.

The centrosome is the major microtubule nucleating and organizing center in mammalian cells, consisting of two cylindrical centrioles, surrounded by multi-layered toroid of pericentriolar matrix (PCM)^1^,^2^. Resting cells contain one centrosome which duplicates strictly once in a cell cycle, synchronized with DNA replication cycle, giving rise to two daughter centrosomes before the onset of mitosis [reviewed in^3^]. Deregulation of the centrosome duplication cycle leads to centrosome amplification, which is commonly observed in multiple human tumors [reviewed in^4^]. Normal cells with supernumerary centrosomes generally die, due to the formation of multipolar spindles leading to severe aneuploidy and prolonged checkpoint arrest and mitotic catastrophe. In contrast, tumor cells with multiple centrosomes are able to cluster centrosomes at opposite poles thus generating pseudo-bipolar spindles. Generation of pseudo-bipolar spindles prevents mitotic catastrophe and promotes limited aneuploidy resulting in an increase in cell survival and also resulting in the generation of invasive tumors^5^,^6^,^7^.

Two centrioles remain closely connected with each other through a proteinaceous linker, during G1^8^. Biogenesis of the nascent daughter centriole (procentriole) begins with the relaxation of the inter-centriolar tether, resulting in separation of the mother centrioles, termed as centriole disjunction^9^,^10^. Centriole disjunction is regulated by orchestrated phosphorylation of various linker proteins including NPM1, β-catenin, Nek2, C-Nap1 (CEP250), rootletin, Cep68, causing their displacement from the linker^11^,^12^,^13^,^14^,^15^,^16^. After steric relaxation, procentriole biogenesis proceeds with step-wise assembly of the central “cart-wheel”^17^,^18^,^19^. Procentrioles mature, through the S and G2 phase, from the proximal end of the mother centriole. If centriole disjunction is blocked, in spite of continued nuclear duplication, centriole duplication remains stalled due to inhibition in cartwheel-templating from mother centriole^20^.

Current studies indicate that, activation of the cdk1/cyclinB complex is involved in generation of altered centrosome number. However, the centrosomal targets of cdk1 and underlying mechanism of cdk1-mediated regulation of centrosome duplication are largely unknown^21^,^22^,^23^,^24^,^25^,^26^. The cdk1/cyclinB1 complex is activated by cdc25C, whose activity is inhibited during interphase by complex formation with 14-3-3 proteins^27^,^28^. Here, we report a novel role of the 14-3-3-protein family^29^ in regulating centrosome number. We demonstrate that, 14-3-3γ and 14-3-3ε localize to the centrosome and control centrosome duplication by preventing premature activation of cdc25C, the cdk1/cyclinB1 complex and the centrosomal protein Nucleophosmin (NPM1)^30^. Loss of 14-3-3γ results in an increase in aneuploidy, cellular transformation and the formation of larger tumors in nude mice. Surprisingly, the expression of a 14-3-3-binding-deficient mutant of cdc25C (S216A) in 14-3-3γ-knockdown cells, at high passage, led to an extensive increase in spindle multi-polarity, a decrease in centrosome clustering, a decrease in cell survival and a reversal of tumor formation in nude mice. These results suggest that 14-3-3γ-mediated premature activation of the mitotic program during interphase results in an induction in spindle multi-polarity, a decrease in centrosome clustering and an inhibition of tumor formation.

## Results

### Loss of 14-3-3γ leads to centrosome amplification

**Figure 1:**
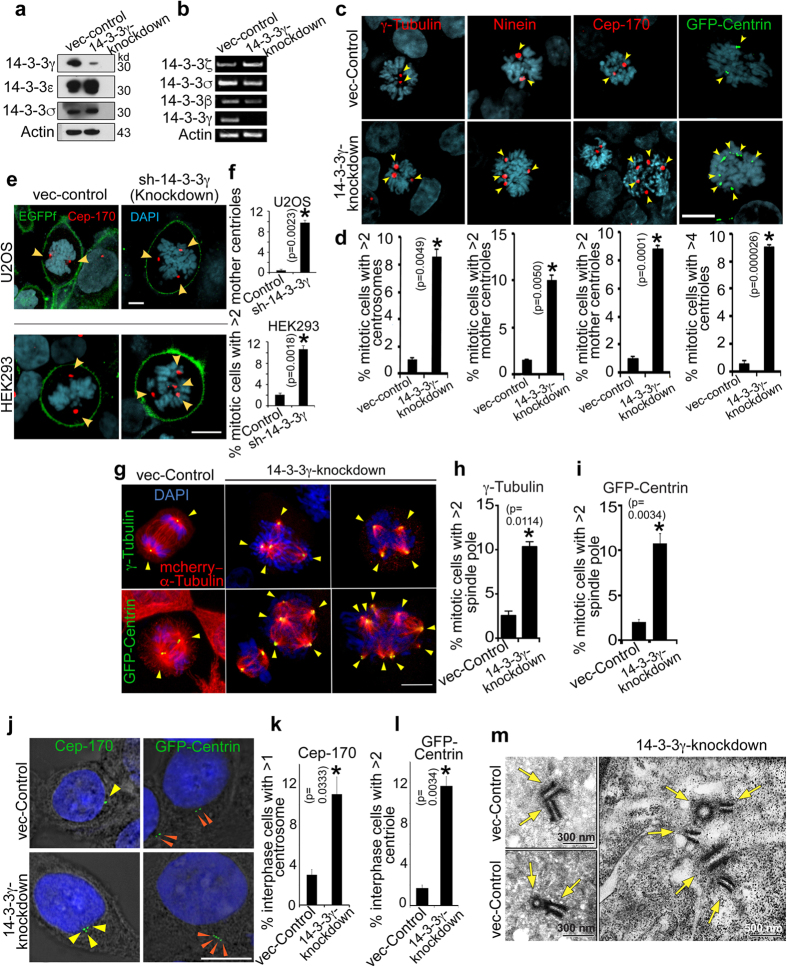
Loss of 14-3-3γ causes centrosome amplification in human cells. (**a–b**) The protein (**a**) and mRNA (**b**) levels of the indicated gene products in 14-3-3γ-knockdown and vector-control cells were determined as described. Actin and GAPDH served as loading controls. (**c–d**) Centrosomes of 14-3-3γ-knockdown and vector-control cells were stained with antibodies specific to γ-Tubulin, Ninein and Cep170, or the cells were transfected with GFP-Centrin as indicated (**c**). Cells were co-stained with DAPI to visualize the nuclei and analyzed by confocal microscopy. Representative images are shown (**c**). The percentage of mitotic cells with more than 2 centrosomes or more than four centrioles was determined in three independent experiments (**d**). (**e–f**) HEK293 or U2OS cells were transfected with plasmids encoding EGFP-f (control) or EGFP-f and shRNA-14-3-3γ. Cells were stained with anti-Cep-170 antibodies, to determine centrosome number. (**g–i**) Multiple spindle poles associated with supernumerary centrosomes were observed by transfecting the cells with mCherry-α-tubulin and GFP-Centrin, or transfection of mCherry-α-tubulin followed by immuno-staining for γ-Tubulin (**g**). The number of spindle poles was determined as indicated in the materials and methods (**h–i**). Note that all the centrosomes anchor the mitotic spindle. (**j–l**) The indicated cells were stained with antibodies to Cep-170 or transfected with GFP-Centrin followed by co-staining with DAPI and the number of centrosomes determined. The images shown are merged with DIC image. (**k**) The number of cells with more than 1 centrosome (for Cep-170) and (**l**) more than 2 centrioles (for Centrin) were determined in three independent experiments. (**m**) Osmium tetroxide stained 14-3-3γ-knockdown and vector-control cells were visualized at 25000x magnification under scanning Electron Microscope. Centrioles are indicated by arrows. All the Western blots were run under the same experimental conditions and the full length blots are in Supplementary Fig. 6. In all the experiments the mean and standard error from at least three independent experiments were plotted, error bars denote standard error of mean and p-values are obtained using Student’s t test (2 sample unequal variance) and the asterisk (*) represents a p value <0.05. Original magnification 630X with 2X optical zoom. Scale bar indicate 10 μm, unless mentioned.

Loss of 14-3-3γ (Fig. 1a,b) results in an override of the S and G2 cell cycle check-points in HCT116 cells, leading to premature mitotic progression^31^. In addition to the loss of checkpoint control, an increase in mitotic index was also observed in 14-3-3γ-knockdown cells (Supplementary Fig. S1a,b), a phenotype often associated with centrosome amplification^32^,^33^. To determine if loss of 14-3-3γ lead to an increase in centrosome number, we determined centrosome number in cells that lack only the 14-3-3γ isoform (Supplementary Fig. S1c and Fig. 1c,d). An increase in number of cells containing supernumerary centrosomes in mitotic phase was observed in the 14-3-3γ-knockdown cells as compared to the vector controls, using antibodies specific to γ-Tubulin [pericentriolar marker^34^], Ninein [mother centriole marker^35^] and Cep-170 [mother centriole marker^36^] (Fig. 1c,d). In addition, cells were also transfected with GFP-Centrin [centriole specific marker^37^] to confirm that loss of 14-3-3γ led to an increase in centrosome number (Fig. 1c,d). Similarly, loss of 14-3-3γ in HEK293 and U2OS cells also led to increased centrosome amplification (Fig. 1e,f). Expression of an shRNA-resistant 14-3-3γ construct resulted in a reversal of centrosome amplification in the 14-3-3γ-knockdown cells (Supplementary Fig. S1d–f), suggesting that centrosome amplification was solely due to loss of 14-3-3γ.

To determine whether the centrosome foci observed in 14-3-3γ-knockdown cells, are intact or fragmented centrosomes, the 14-3-3γ-knockdown and vector control cells were transfected with GFP-Centrin followed by immuno-staining with antibodies to Pericentrin^38^. All the amplified centrosome foci stained for both the centrosomal markers, thus ruling out the possibility that the structures we observe upon 14-3-3γ-loss are centrosome fragments (Supplementary Fig. S1g,h). To determine whether the additional centrosomes can anchor spindles, 14-3-3γ-knockdown cells were transfected with mCherry-α-Tubulin and GFP-Centrin or stained with antibodies to γ-Tubulin, followed by confocal microscopy. All the centrosomes present in 14-3-3γ-knockdown cells act as spindle generation centers (Fig. 1g–i). A minor increase in protein levels was observed for the centrosome protein required for cartwheel formation, h-SAS6 and an increase in the activation of the centrosome associated kinase Aurora A was observed upon loss of 14-3-3γ (Supplementary Fig. S5a,b).

**Figure 2:**
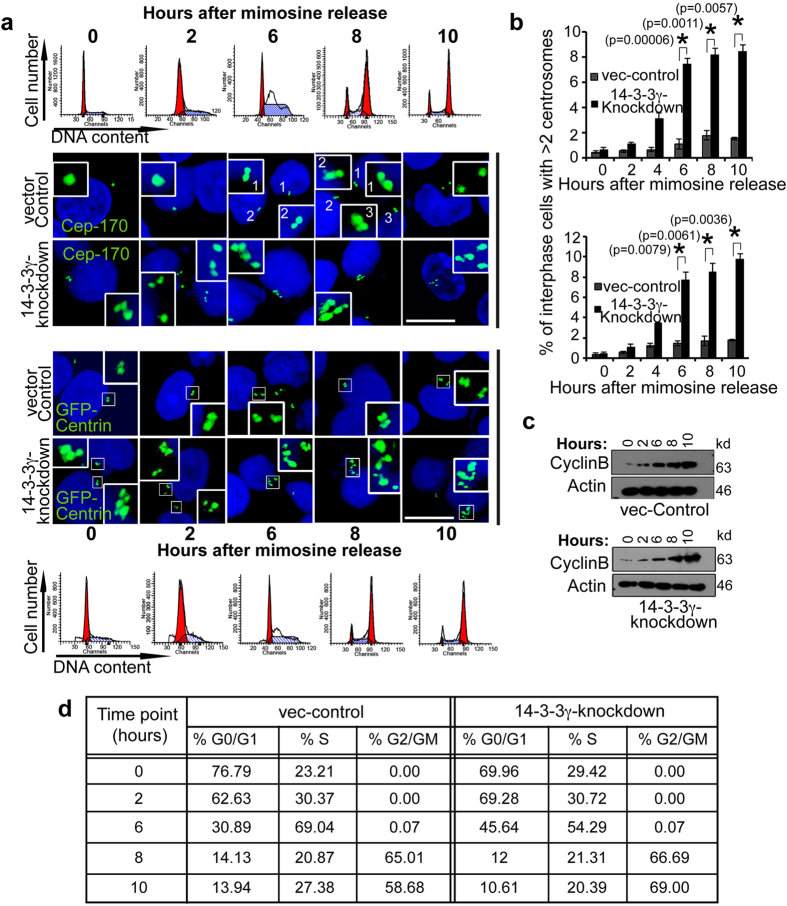
Depletion of 14-3-3γ leads to centrosome over-duplication during S-phase. (**a,b**) 14-3-3γ-knockdown and vector-control cells were transfected with GFP-Centrin and synchronized with mimosine. Another similar set of un-transfected cells were used for staining with anti-Cep-170 antibody. At various time points post mimosine withdrawal (0 hours), cells were either processed for FACS analysis, or were stained with anti-Cep-170 antibody to visualize centrioles and co-stained with DAPI for nuclei. The cells transfected with GFP-Centrin were counter-stained with DAPI. The cell cycle histograms and associated representative confocal images are shown (**a**). The percentage of cells with centrosome amplification was determined at each time point in each experiment and the mean and standard error are plotted (**b**); Student’s t test (2 sample unequal variance) was used to determine p-value; p < 0.05 (*). (**c**) Protein extracts were resolved on SDS-PAGE gels followed by Western blotting for cyclinB1. Note that an increase in CyclinB1 level is coincident with an increase in centrosome number. Western blots for actin served as a loading control. (**d**) Table showing the percentages of cells in different cell cycle phases at each time point. All the Western blots were run under the same experimental conditions and the full length blots are in Supplementary Fig. 6.

To determine if the increase in centrosome number is observed in other cell cycle phases, centrosome number was estimated in interphase cells. An increase in centrosome number during interphase was also observed in 14-3-3γ-knockdown cells as compared to the vector control, using immunofluorescence with antibodies to Cep-170 and Centrin (Figs 1j–l and 2). To further confirm that we were observing intact centrioles, centrioles were examined using transmission electron microscopy. 14-3-3γ-knockdown cells showed the presence of more than two centrioles during interphase in comparison to the vector-control cells (Fig. 1m).

### Centrosome over-duplication is the cause of centrosome amplification

Centrosome amplification can occur in two ways — (i) by *de novo* synthesis without using an existing centriole as template or, (ii) due to defects in template dependent centriole duplication process, which is termed as centriole over-duplication or hyper-duplication^39^,^40^. To determine the time-point in the cell cycle at which centrosome amplification takes place, cells were synchronized in late G1 with mimosine, prior to the initiation of centrosome duplication, released from the mimosine block and then followed over time. We have quantitated the number of cells with >2 centrosomes in these figures so as to provide a comparison with the experiments shown in Fig. 1. We have not determined the number of cells with only one centrosome, which would be the standard centrosome number during G1 and early S phase. The quantitation shown in this figure does not take into account the number of centrosome amplification events that have occurred in the previous cycle and therefore, the increase in centrosome number upon loss of 14-3-3γ is not observed at early time points in the cell cycle. An alternative explanation is that cells with multiple centrosomes demonstrate an increased sensitivity to mimosine. However, it was observed that centrosome number increases during S-phase between 6–10 hours post release from mimosine and coincides with an increase in the expression of cyclinB1 (Fig. 2a–d). Thus, centrosome amplification, caused by the loss of 14-3-3γ occurs due to centriole over-duplication during interphase.

### 14-3-3γ-mediated centrosome over-duplication leads to increased aneuploidy and early tumor formation

**Figure 3:**
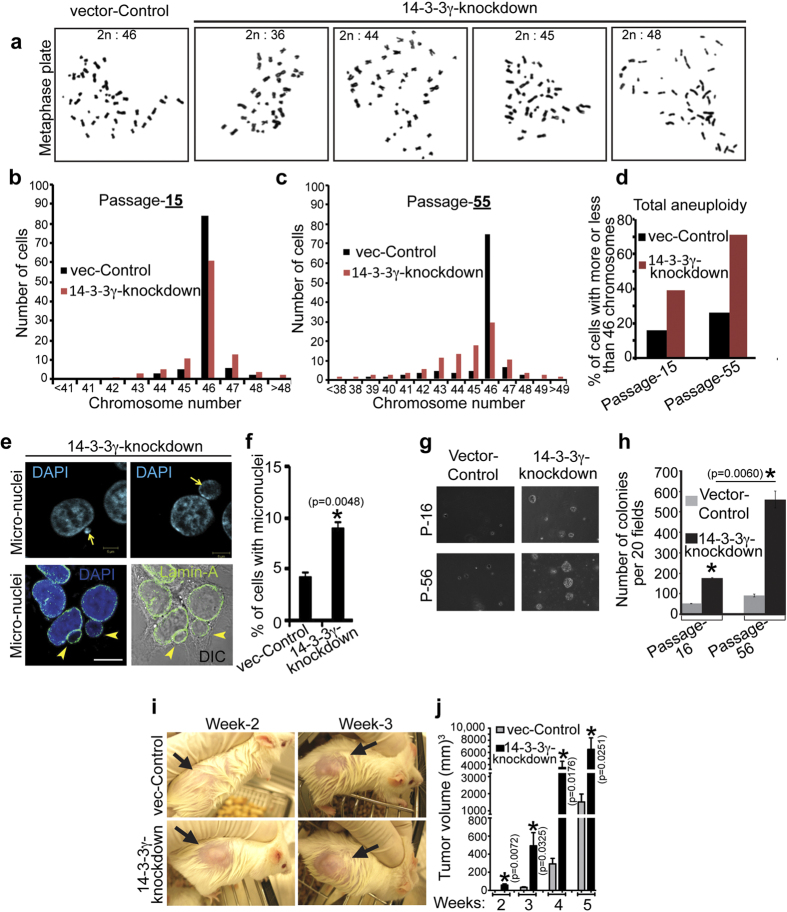
Centrosome amplification, resulted by depletion of 14-3-3γ, leads to higher aneuploidy and increased tumor formation. (**a–d**) Chromosome counts from 14-3-3γ-knockdown cells show an increase in aneuploidy in comparison to the vector control cells. (**a**) Representative metaphase plates (visualized under 1000x magnification of upright fluorescence microscope) with different chromosome numbers from 14-3-3γ-knockdown and vector control cells are shown. (**b,c**) Chromosome numbers from one hundred 14-3-3γ-knockdown and vector control cells were counted at early (15) or late (55) passage. Aneuploidy increases with loss of 14-3-3γ and rise in cellular passage. (**d**) Difference in overall aneuploidy (total number of cells with more or less than 46 chromosomes) between knockdown and control line, at passage-15 and 55, is plotted. (**e**) Micronuclei were observed by DAPI staining or transfection with GFP-Lamin-A. Scale bar is 10 μm unless mentioned. (**f**) The number of cells showing micronuclei formation was determined in the 14-3-3γ-knockdown and vector control cells. 100 cells were counted in three independent experiments and the mean and standard error are plotted. (**g,h**) Early and late passage 14-3-3γ-knockdown and vector control cells were plated in soft agar and colonies counted formed after 2-3 weeks from 20 fields (at 10X magnification) and the mean and standard deviation from three independent experiments is plotted. (**i**) 5 NOD-SCID mice were subcutaneously injected with 10^6^ cells of 14-3-3γ-knockdown and control line, and tumor size determined every week as described. (**J**) Tumor volume is plotted on the Y-axis and the time in weeks on the X-axis. The corresponding mean tumor volumes in mm^3^ are: Week 2 vec-Control 0, 14-3-3γ knockdown 58.8; Week 3 vec-Control 33.5, 14-3-3γ knockdown 492.5; Week 4 vec-Control 293, 14-3-3γ knockdown 3510; Week 5 vec-Control 1,519, 14-3-3γ knockdown 6,497. An asterisk (*) indicates a p value <0.05. p-values were determined using a Students t-test (2 sample unequal variance).

Cells with multiple spindle poles either die due to a failure to complete mitosis, or survive after a multi-polar mitosis. On occasion, these cells cluster multiple centrosomes at two spindle poles leading to (i) an increase in aneuploidy, (ii) a favorable microtubule organization and (iii) often tumor progression^39^. To test if centrosome over-duplication leads to increased aneuploidy, chromosome number was determined in the 14-3-3γ-knockdown and vector-control cells by counting chromosomes in metaphase plates; HCT116 cells are near diploid and have a normal chromosomal complement (ATCC). Loss of 14-3-3γ-led to an increase in the number of cells with more or less than 46 chromosomes as compared to the vector control (Fig. 3a,b). Moreover, the extent of aneuploidy was increased with sub-culturing (“passage”) of the 14-3-3γ-knockdown cells (Fig. 3c,d); cells in passage-55 showing a greater increase in aneuploidy as compared to cell in passage-15. Aneuploidy leads to chromosome instability and chromosome lagging during anaphase, which often results in cells containing micronuclei that appear as extra-nuclear satellite around the nucleus^41^,^42^. An increase in micronuclei formation was observed in 14-3-3γ-knockdown cells by staining with nuclear specific stain DAPI. Micronuclei also remain surrounded by nuclear membrane, which was observed with antibody specific to nuclear membrane protein, Lamin-A (Fig. 3e,f).

As increase in aneuploidy often leads to neoplastic transformation, soft agar assays were performed to determine if loss of 14-3-3γ could lead to an increase in cellular transformation. 14-3-3γ-knockdown cells showed significantly higher number of colonies with increased diameter in soft agarose assay, in comparison to the control line (Fig. 3g,h and Supplementary Fig. S1i). The number of colonies formed by the 14-3-3γ-knockdown cells showed a greater increase with an increase in passage as compared to the vector control cells. The 14-3-3γ-knockdown cells develop tumors at an early time point and form larger tumors in immune-deficient (NOD-SCID) mice, in comparison to the vector-control cells (Fig. 3i,j). The tumors formed by 14-3-3γ-knockdown cells showed an increased centrosome number in comparison to the tumor tissue sections of vector-control (Supplementary Fig. S1j).

### 14-3-3γ localizes to the centrosome and interacts with centrosomal proteins

**Figure 4:**
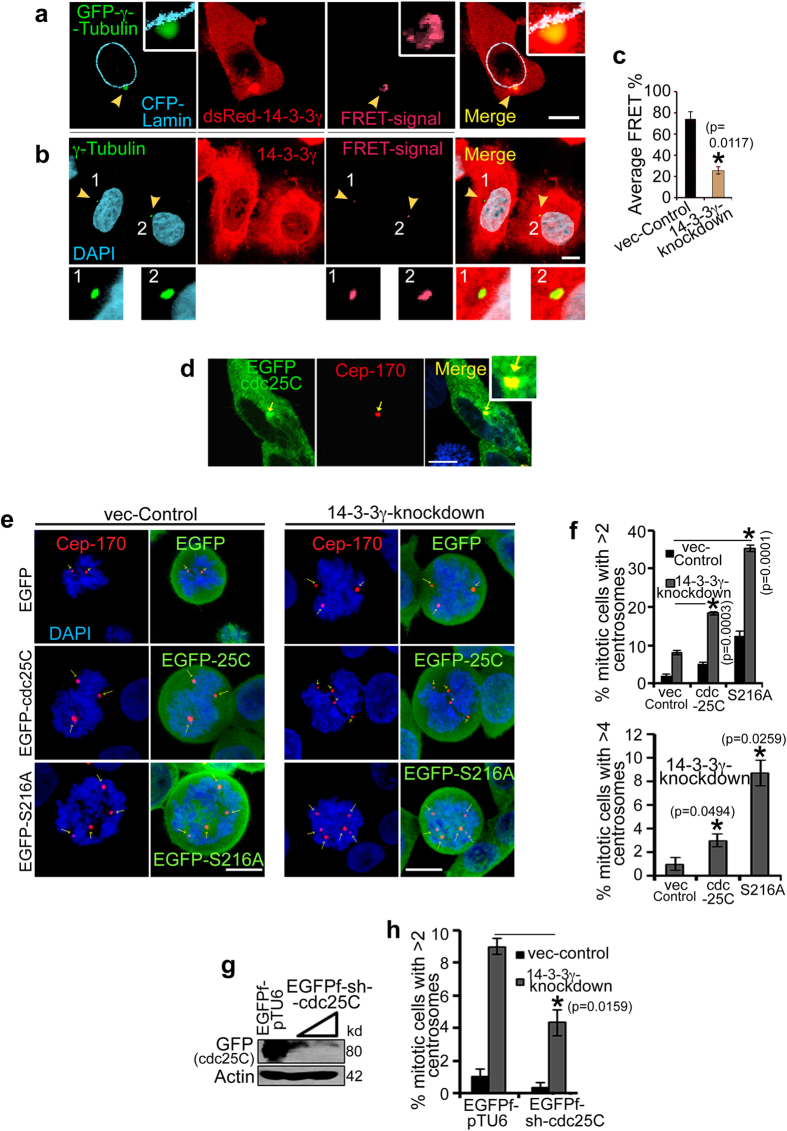
Cdc25C activity stimulates centrosome over-duplication in 14-3-3γ-knockdown cells. (**a–c**) HCT116 cells were transfected with CFP-Lamin-A (cyan), GFP-γ-Tubulin (donor) and dsRed-14-3-3γ (acceptor) (**a**) or stained with antibodies to γ-Tubulin (green) and 14-3-3γ (red) (**b**) and counter-stained with DAPI (blue) followed by sensitized emission FRET analysis. The FRET images are shown in the third panel from the left, while the fourth image from the left shows the merged image as indicated. The smaller panels show a magnified image of the boxed region. (**c**) A comparison of FRET signal intensities obtained from antibody based FRET was performed in 14-3-3γ-knockdown and control line. The graph shows the mean and standard deviation for percentage FRET efficiency from ten different cells. p values indicated were obtained using a student’s t-test (2 sample unequal variance) and the asterisk indicates p < 0.05. (**d**) Co-localization of cdc25C at centrosome is determined by transfecting HCT116 cells with EGFP-cdc25C (green) and staining with anti Cep-170 (red) antibody. (**e–g**) 14-3-3γ-knockdown cells were transfected with the vector control (EGFP), EGFP-cdc25C or EGFP-S216A and stained with antibodies to Cep-170 (red) (**e**). (**f,g**) Centrosome number was determined and the mean and standard error from three different experiments is plotted (**h**) 14-3-3γ-knockdown and control cells were co-transfected with GFP-cdc25C and increasing concentration of EGFPf-sh-cdc25C. Western blot is showing the reduction in GFP-cdc25C upon expression of sh-cdc25C. (**i**) Vector control and 14-3-3γ knockdown cells were transfected with the vector control (EGFPf-pTU6) or EGFP-f-shcdc25C. The transfected cells were stained with antibodies to Cep170 and centrosome number was determined in a 100 transfected cells (identified by GFP expression) in three independent experiments. In all cases p values were obtained using a student’s t-test. Scale bars are 10 μm unless mentioned. All the Western blots were run under the same experimental conditions and the full length blots are in Supplementary Fig. 6.

To determine whether 14-3-3γ localizes to the centrosome and forms complex with centrosomal proteins, we performed sensitized emission fluorescence resonance energy transfer (FRET) assays, as described in the materials and methods section^43^. FRET analysis using fluorescently tagged versions of 14-3-3γ and γ-Tubulin demonstrated that 14-3-3γ and γ-Tubulin are in close physical proximity at the centrosome (Fig. 4a). FRET analysis, using immuno-staining with antibodies specific to 14-3-3γ and γ-Tubulin, showed a significantly reduced FRET signal from the 14-3-3γ-knockdown cells as compared to the vector controls, suggesting that 14-3-3γ binds to γ-Tubulin and localizes to the centrosome (Fig. 4b,c). As 14-3-3γ often forms a dimer with 14-3-3ε^44^, we determined if 14-3-3ε localized to the centrosome using the PCM marker γ-Tubulin. Confocal microscopy demonstrated that 14-3-3ε localized to the centrosome, however, no FRET was observed between 14-3-3ε and γ-Tubulin (Supplementary Fig. S2d). Similarly, biochemical analyses using bacterially purified 14-3-3 proteins or co-immunoprecipitation assays demonstrated that 14-3-3γ forms a complex with γ-Tubulin, whereas 14-3-3ε showed very little to no interaction with γ-Tubulin (Supplementary Fig. S2e,f). Few other putative centrosomal interactors of 14-3-3γ such as, GCP2^45^, KIF5B^46^, KLC2^47^ were also identified in GST-pulldown coupled mass spectrometric (MALDI-TOF) analysis (Supplementary Fig. S2g). As 14-3-3γ and 14-3-3ε localize to the centrosome, we tested the effect of combined loss of both the isoforms on centrosome amplification. Expression of both 14-3-3γ and 14-3-3ε was inhibited using vector driven RNAi and centrosome number was determined in mitotic cells. Loss of either 14-3-3ε or 14-3-3γ led to a similar increase in centrosome number in HCT116 cells, in comparison to the vector control (Supplementary Fig. S2a–c). However, inhibiting the expression of both 14-3-3γ and 14-3-3ε resulted in an additive effect in the increase in centrosome number. Therefore, these results suggest that 14-3-3ε and 14-3-3γ localize to the centrosome and prevent centrosome re-duplication in HCT116 cells.

### 14-3-3γ prevents centrosome amplification by inhibiting cdc25C function

14-3-3γ and 14-3-3ε form a complex with cdc25C and resulting in an inhibition of cdc25C function by preventing it from activating the substrate cdk1/cyclinB^27^,^48^,^49^. Cdc25C localizes to centrosome^50^,^51^ and activates the centrosomal cdk1/cyclinB1 complex, resulting in activation of the mitotic cascade^52^,^53^. Therefore it is possible that increased cdc25C activation, upon loss of 14-3-3γ, could be responsible for centrosome amplification in the 14-3-3γ knockdown cells. As previously reported by this laboratory^31^, immuno-blotting with phospho-S216-cdc25C specific antibodies demonstrate that cdc25C is constitutively active in the 14-3-3γ knockdown cells due to a decrease in the levels of cdc25C phosphorylated on S216 when compared to the vector control (Supplementary Fig. S3a). GFP-tagged cdc25C co-localizes with the centrosome during interphase as demonstrated by immuno-staining with antibodies to Cep170 (Fig. 4d). Over-expression of cdc25C or the 14-3-3-binding-defective mutant cdc25C-S216A in 14-3-3γ-knockdown cells resulted in a greater increase in centrosome amplification than that observed with the vector control cells (Fig. 4e,f and Supplementary Fig. S3b) and the increase was similar to that observed when the expression of both 14-3-3γ and 14-3-3ε were inhibited in these cells. Inhibition of cdc25C expression using vector driven RNAi reduced the extent of centriole over duplication (Fig. 4g,h and Supplementary Fig. S3c). These results suggest that an increase in cdc25C activity leads to centrosome over-duplication. As other cdc25 isoforms also form a complex with 14-3-3 proteins though not with 14-3-3γ^54^,^55^, the effect of over-expressing cdc25A and cdc25B on centrosome number was determined in the vector control and 14-3-3γ-knockdown cells. Epitope tagged cdc25A, cdc25B and cdc25C were transfected into the vector control and 14-3-3γ-knockdown cells and centrosome number determined as described in materials and methods. Expression of the individual cdc25 isoforms resulted in 10–20% increase in 14-3-3γ-knockdown cells containing multiple centrosomes, in comparison to the vector-control cells (Supplementary Fig. S3e). Similarly, a knockdown of cdc25A, B and C by individual shRNA constructs reduced the percentage of multiple centrosome containing 14-3-3γ-knockdown cells (Supplementary Fig. S3f). These results suggest that all the cdc25 isoforms contribute to centrosome amplification in this cell type.

### Premature activation of cdk1 during interphase results in centrosome amplification

**Figure 5:**
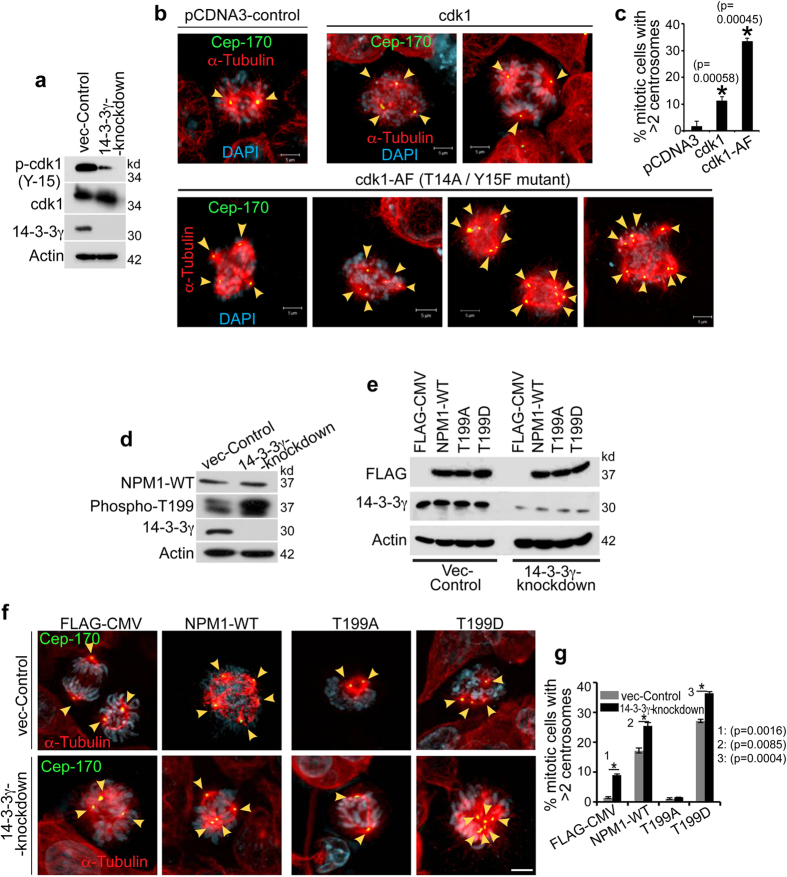
Increased NPM1 phosphorylation at T199 leads to centrosome duplication in 14-3-3γknockdown cells. (**a**) Protein extracts from vector control or 14-3-3γ knockdown cells were resolved on SDS-PAGE followed by immuno-blotting with the indicated antibodies. Actin served as loading control. (**b,c**) HCT116 cells were transfected with mCherry-α-tubulin and co-transfected with either the control plasmid (pCDNA3) or plasmids encoding wild type cdk1 (cdk1) or constitutively active cdk1 (cdk1AF). Post-transfection the cells were stained with antibodies to Cep170 (green) and DAPI (blue) (**b**). The percentage of mitotic cells with >2 centrosomes was determined in three independent experiments (**c**). (**d**) Protein extracts from the vector control and 14-3-3γ knockdown cells were resolved by SDS-PAGE, followed by Western blotting with the indicated antibodies. Note that NPM1 is phosphorylated on T199 to a greater degree in the 14-3-3γ knockdown cells. Actin served as loading control. (**e**) The 14-3-3γ -knockdown and vector control cells were transfected with either the vector control or FLAG-epitope tagged versions of WT NPM1 or the NPM1 mutants (T199A and T199D). Post transfections, protein extracts prepared from these cells were resolved by SDS-PAGE followed by Western blotting with the indicated antibodies. Western blots for actin serves as loading controls. (**f,g**) 14-3-3γ-knockdown and vector-control cells, transfected with mCherry-α-tubulin and FLAG-NPM1 constructs, were stained with antibodies to Cep-170, co-stained with DAPI and followed by confocal microscopy (**f**) to determine the percentage of cells containing >2 centrosomes. The mean and standard deviation of three independent cells is plotted (**g**) p values were determined using a Student’s t-test (2 sample unequal variance) with p < 0.05. Scale bars indicate 5 μm. All the Western blots were run under the same experimental conditions and the full length blots are in Supplementary Fig. 6.

Cdc25C activates cdk1/CyclinB1, by dephosphorylating Thr14 and Tyr15 residues of cdk1 [reviewed in^56^] and therefore we determined if the increase in centrosome amplification observed upon cdc25C activation was due to an increase in cdk1 activity. A reduction in Tyr-15 phosphorylation of cdk1 in 14-3-3γ-knockdown cells indicated that cdk1 was active in 14-3-3γ-knockdown cells (Fig. 5a). If cdk1 activation is responsible for the centrosome over-duplication upon loss of 14-3-3γ, over-expression of cdk1 or the constitutively active mutant, cdk1AF^57^, should result in an increase in centrosome number. Over-expression of either cdk1 or cdk1-AF resulted in an increase in centrosome over-duplication in HCT116 cells, a phenotype similar to that observed upon the over-expression of cdc25C (Fig. 5b,c and Supplementary Fig. S4a,b). The increase in centrosome duplication by over-expression of cdk1-AF was comparable to that observed with cdc25C-S216A expression in 14-3-3γ-knockdown cells (Figs 4g and 5c). Inhibition of cdk1 expression reduced centrosome amplification in 14-3-3γ-knockdown cells (Supplementary Fig. S4c,d). Similar results were obtained when we inhibited the expression of cdk2, which has previously been shown to be essential for centrosome duplication^11^. Depletion of both the cdks resulted in an additive decrease in centrosome duplication. These results suggest that the presence of an active cdk1 complex is responsible for centrosome over-duplication and are consistent with our data that centrosome over-duplication is co-incident with the expression of CyclinB1 in these cells (Fig. 2d).

### Cdk1 phosphorylates the centriolar linker protein NPM1 leading to centrosome amplification

Phosphorylation of the inter-centriolar linker protein Nucleophosmin (or NPM1, also known as B23, Numatrin or NO38) at T199 residue causes its detachment from the centriolar linker, thus increasing the distance between two centrioles and providing the spatial signal for procentriole biogenesis, templating from the mother centriole^16^,^58^,^59^. *In vitro* kinase assays demonstrated that the T199 residue of NPM1 could be phosphorylated by cdk1/cyclinB1 complex, while the phospho-deficient mutant, NPM1-T199A does not serve as an efficient substrate for cdk1 (Supplementary Fig. S4e,f). In addition, an increase in T199 phosphorylation of NPM1 was also observed in the 14-3-3γ-knockdown cells upon immuno-blotting with phospho-specific antibodies recognizing NPM1 phosphorylated on T199 (Fig. 5c). To test the effect of cdk1-mediated T199-phosphorylation of NPM1 on centrosome over-duplication, we expressed the phospho-mimetic (T199D) and phospho-deficient (T199A) mutants of NPM1 in 14-3-3γ-knockdown cells (Fig. 5d). The extent of centrosome amplification was significantly reduced with the expression of NPM1-T199A, while the centrosome amplification was increased upon T199D expression (Fig. 5e,f). To confirm that the increase in NPM1 phosphorylation occurs during S-phase, a cell cycle synchrony experiment was performed as described earlier. Western blot analyses demonstrated that NPM1 phosphorylation in the vector control cells first appears at 10 hours post release from mimosine when the majority of the cells are in G2 phase, while in the 14-3-3γ knockdown cells NPM1 phosphorylation appears at the six hour time point and coincides with the increased expression of cyclin B1 (Supplementary Fig. S5c). These results are consistent with our observation that cdk1 is prematurely active upon loss of 14-3-3γ (Fig. 5a), suggesting that cdk1 might be the major NPM1 kinase in cells lacking 14-3-3 γ. Therefore, premature activation of cdk1 in 14-3-3γ-knockdown cells causes hyper-phosphorylation of T199 residue of NPM1 and possibly other centrosomal proteins resulting in centrosome amplification.

### Premature cdc25C activation reduces centrosome clustering and inhibits tumor growth

**Figure 6:**
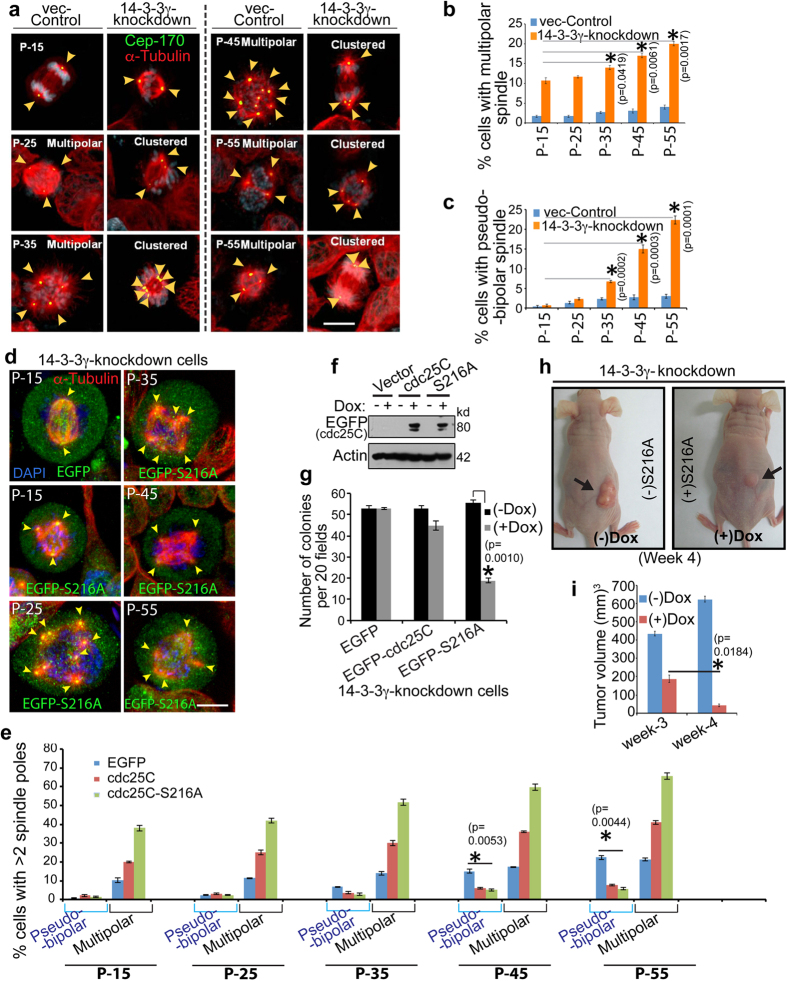
Over-expression of cdc25C-S216A leads to an increase in multipolar mitoses and a decrease in neoplastic progression. (**a**) 14-3-3γ-knockdown and vector control cells at different passages (P) were fixed and stained with antibodies to α-tubulin and Cep-170 and co-stained with DAPI. Arrows indicate centrosome poles. (**b,c**) The percentage of cells with multi-polar spindles and pseudo bi-polar spindles was determined in three independent experiments and the mean and standard error plotted. (**d**) 14-3-3γ-knockdown cells were transfected with EGFP, EGFP-cdc25C or EGFP-S216A constructs. Cells were fixed and stained with anti-α-Tubulin antibody and co-stained with DAPI. Arrows indicate spindle poles. (**e**) The percentage of cells with multi-polar spindles and pseudo bi-polar spindles was determined in three independent experiments and the mean and standard deviation plotted. A significant increase in the number of cells with multi-polar spindles and a corresponding decrease in the number of cells with pseudo-bipolar spindles was observed in cells expressing the two cdc25C constructs at all passages. (**f,g**) 14-3-3γ-knockdown cells were transfected with doxycycline (Dox) inducible constructs expressing either GFP or EGFP-cdc25C or EGFP-cdc25C-S216A. Post transfection the cells were selected in puromycin to select transfected cells. Transfected cells were grown in the presence or absence of doxycycline and protein extracts were resolved on SDS-PAGE gels followed by Western blotting with the indicated antibodies (**f**) or the cells were embedded in soft agar in the presence or absence of doxycycline and colony formation determined in three independent experiments. The mean and standard deviation are plotted (**g**). (**h,i**) 14-3-3γ-knockdown cells were transfected with doxycycline (Dox) inducible constructs expressing EGFP-cdc25C-S216A. Post selection, cells were injected subcutaneously in Nude mice. One set was given doxycycline in the drinking water (+Dox) and the other set received no doxycycline (−Dox). Tumor volume was measured and the mean and standard error are plotted from 4 × 2 sets of mice at different times post injection. p values were obtained using Student’s t test (2 sample unequal variance). p values <0.05 are indicated by asterisk. All the Western blots were run under the same experimental conditions and the full length blots are in Supplementary Fig. 6.

Normal cells cannot tolerate centrosome amplification and die eventually due to spindle asymmetry, mitotic catastrophe, unfavorable aneuploidy or defects in interphase cytoskeletal organization^60^,^61^. In contrast, aggressive tumor cells have evolved a mechanism to cluster multiple centrosomes and thus generating a pseudo-bipolar spindle during mitosis to maintain a lower level of aneuploidy thus preventing mitotic catastrophe^62^,^63^,^64^. Tumorigenic potential and aggressiveness of cultured cells generally increase with the increase in sub-culturing or “passage”^65^,^66^. A gradual increase in centrosome clustering with progressive sub-culturing was observed in the 14-3-3γ-knockdown cells as compared to the vector control (Fig. 6a–c). While an increase in multi-polar spindle formation was also observed in 14-3-3γ-knockdown cells, the increase in the number cells with clustered centrosomes is greater in the 14-3-3γ knockdown cells. This suggests that loss of 14-3-3γ leads to the selection of a population of cells that are capable of clustering their supernumerary centrosomes.

**Figure 7:**
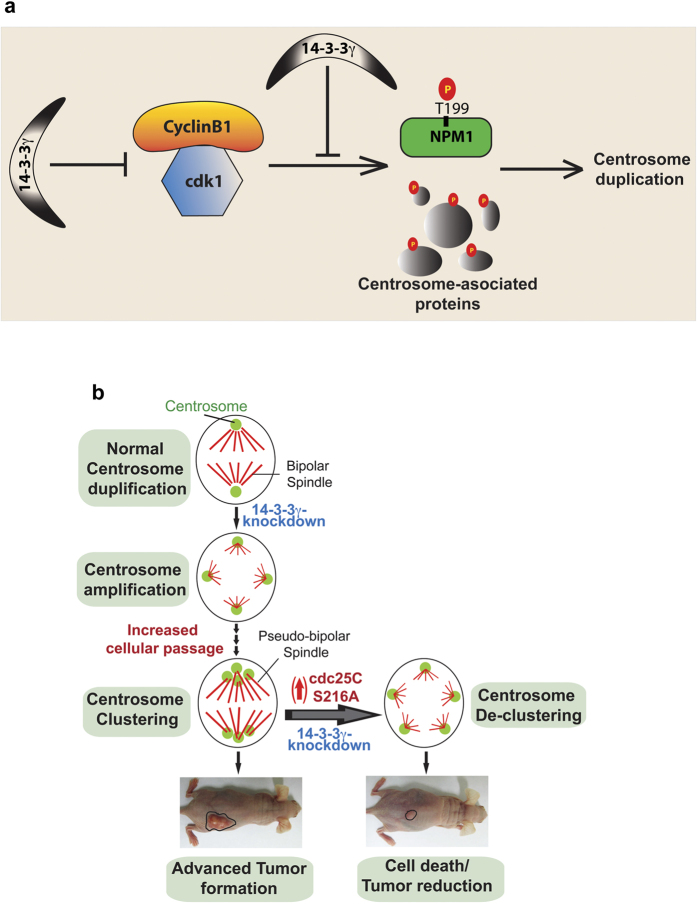
Model for centrosome duplication and reduced tumor formation upon activation of cdc25C. (**a**) 14-3-3γ sequesters cdc25C in cytoplasm during S phase and thus cdk1 remains inactive due to the inhibitory phosphorylation at Thr-14 and Tyr-15. As a consequence, NPM1 is not phosphorylated at T199 and remains associated with inter-centriolar linker. (**b**) Loss of 14-3-3γ causes activation of cdc25C resulting in dephosphorylation and activation of cdk1. Active cdk1 phosphorylates T199 of NPM1. T199 phosphorylation of NPM1 results in its dissociation from centriolar linker. Dissociation of NPM1 from centriolar linker leads to centriole separation (disjunction) and thus provides steric permission for procentriole nucleation and maturation. (**c**) Expression of active-cdc25C (cdc25C-S216A), in 14-3-3γ-knockdown cells prematurely activates cdk1, which leads to centrosome hyper-duplication through increased phosphorylation of residue T199 in NPM1. (**d**) Centrosomes cluster with the increase in passage in the 14-3-3γ knockdown cells leading to increased transformation. A decrease in centrosome clustering is observed upon premature activation of cdc25C, resulting in a decrease in tumor growth.

Inhibition of centrosome clustering has been shown to result in tumor cell death by generating multipolar mitoses, or by leading to abnormalities in cell polarization, focal adhesion and migration^64^,^67^. As over-expression of cdc25C in the 14-3-3γ-knockdown cells led to an increase in centrosome number, we wished to determine if the increase in centrosome number affected cell survival and tumor formation. MTT assays demonstrated that expression of both WT cdc25C and the cdc25C-mutant (S216A) in the 14-3-3γ-knockdown cells resulted in a significant decrease in cell viability (Supplementary Fig. S3d). We tested if over-expression of cdc25C-S216A in 14-3-3γ-knockdown cells could reverse centrosome clustering observed in the 14-3-3γ knockdown cells. Expression of either cdc25C or cdc25C-S216A in 14-3-3γ-knockdown cells at different passages led to a decrease in centrosome clustering and increase in spindle-multipolarity (Fig. 6d,e). To determine if this decrease in centrosome clustering and cell viability is associated with a decrease in neoplastic transformation, doxycycline inducible constructs for cdc25C or cdc25C-S216A were expressed in the 14-3-3γ-knockdown cells (Fig. 6f). Cells were selected in puromycin and soft agar assays performed in the presence or absence of doxycycline. A significant reduction in soft agar colony formation was observed in 14-3-3γ-knockdown cells expressing cdc25C-S216A (Fig. 6g). To find if over-expression of cdc25C-S216A in the 14-3-3γ-knockdown cells could lead to a decrease in tumor formation, 14-3-3γ-knockdown cells were transfected with the inducible constructs of cdc25C-S216A described above. 48 hours post transfection cells were enriched by puromycin selection and then injected subcutaneously into nude mice. The mice were segregated into two groups and one group was given doxycycline in the drinking water to induce S216A expression. Mice given doxycycline in the drinking water developed smaller tumors than mice that were not given doxycycline, suggesting that cdc25C-S216A expression leads to a decrease in tumor formation (Fig. 6h,i). Therefore we conclude that, depletion of 14-3-3γ and the premature hyper-activation of cdc25C, causes hyper-activation in cdk1 and hyper-phosphorylation of NPM1 at T199 residue and other centrosome associated proteins (Fig. 7a) during interphase leading to (i) reversal of centrosome clustering, (ii) generation of extensive spindle multipolarity, (iii) cell death in culture and (iv) inhibition of tumorigenesis in mice (Fig. 7b).

## Discussion

We report for the first time that, loss of 14-3-3γ causes centrosome over-duplication, centrosome clustering and tumor formation in mice. Although 14-3-3 proteins have been isolated from centrosomal fractions^68^, the molecular basis and isoform specific role of 14-3-3 in centrosome duplication remained unknown. In this study, we have demonstrated that 14-3-3ε and 14-3-3γ localize to the centrosome and form complexes with centrosomal proteins. Our results demonstrate a mechanism by which 14-3-3γ restricts centrosome duplication to once per cell cycle, by inhibiting cdc25C function, thus preventing premature activation of cdk1 during interphase and resulting in a decrease in phosphorylation of T199 residue of the centriolar linker protein NPM1. Identification of the molecular basis of 14-3-3γ-mediated centrosome duplication helped to design a way to reduce centrosome clustering, leading subsequently to a decrease in tumor formation in nude mice by over-expression of constitutively active (14-3-3-binding-deficient) cdc25C in 14-3-3γ-knockdown cells. This also suggests that complete disruption of the cdc25C-14-3-3 complex during interphase might be a way to inhibit tumor growth and selective tumor cell killing.

In *S. pombe*, cdc25 activates the cdc2 (cdk1-ortholog)/cyclinB complex, which phosphorylates a spindle pole body protein Cut12 (Stf1). Gain of function mutation in Cut12 is sufficient to drive mitosis in the absence of cdc25C, and loss of Cut12 results in the failure in microtubule nucleation, failure to associate Spindle Pole Bodies to the nuclear membrane and failure in mitotic entry leading to mitotic arrest. The defects observed upon Cut12 deletion could be rescued by enhancing cdc25C or cdk1 function suggesting that cdc25C and cdk1 have a role to play in centrosome duplication^24^,^69^,^70^. However, experiments in other cell systems have suggested that loss of cdk1 leads to an increase in centrosome number due to the induction of multiple rounds of S-phase^71^,^72^. One of these studies^71^ reported that, while inhibition of cdk1 in rodent cells causes centrosome amplification due to endo-reduplication, the same phenotype was not observed in human cell lines including HCT116 and U-2OS, similar to the results reported here. The differences observed between experiments performed in *Drosophila* or rodent cell lines in culture and in human cell lines might be due to species specific differences in centrosome duplication. Altogether, these observations support the fact that increased cdk1-activation can lead to centrosome amplification in human cells. Cdk1 overexpression leads to genomic instability, centrosome separation^22^,^23^,^73^ and centrosome amplification^74^. Nam and van Deursen recently showed that cyclin B overexpression in the mouse leads to accelerated centrosome separation, chromosome mis-segregation and tumor formation through a Plk1−Nek2−C-NAP1/rootletin mediated pathway^26^,^75^. Therefore, it is evident that increased activation of the cyclin B/cdk1 complex leads to centrosome amplification, aneuploidy and tumor formation. Further, activation of cdk1 leads to the activation of Plk1 by the Aurora-A and Bora kinases^76^. Plk1 activation is responsible for Cep-152/Cep-192 mediated activation of Plk-4, which drives centriolar cartwheel formation through 9-fold assembly of Sas-6 dimers^77^,^78^,^79^. Thus, a positive feedback loop of cdk1 is required for the commitment of M phase progression^80^ and initiation of centriole biogenesis.

Although NPM1 is phosphorylated by the G1/S checkpoint regulator cdk2^16^, it was not clear if NPM1 could also serve as a cdk1 substrate for centriole disjunction during S-phase^12^,^59^. In this study, we have demonstrated that T199 residue of NPM1 is a target of cdk1, and early phosphorylation of NPM1 by cdk1 leads to a premature increase in centrosome number. Further, our results suggest that in HCT116 cells cdk1 is the major NPM1 kinase and that the increase in NPM1 phosphorylation upon loss of 14-3-3γ is due to a premature increase in cdk1 activity. Our data does not exclude the possibility that cdk1 phosphorylates other centrosomal proteins, in addition to NPM1, and that these substrates might also contribute to the increase in centrosome number observed upon loss of 14-3-3γ (Fig. 7a). Interestingly, loss of either cdk1 or cdk2 in the 14-3-3γ knockdown cells leads to a decrease in centrosome number, suggesting that cdk1 is not phosphorylating the same set of substrates as cdk2. Therefore, when cdk1 is prematurely activated during S-phase in the 14-3-3γ-knockdown cells, it stimulates centrosome over-duplication and this requires cdk2 activity suggesting that the activity of both proteins is required for centrosome amplification.

Our work also demonstrates that the mitotic phosphatase cdc25C is required for the increase in centrosome number observed upon loss of 14-3-3γ. However, over-expression of all the cdc25 isoforms in HCT116 cells results in an increase in centrosome number and a knockdown of the individual isoforms reduces centrosome number (Supplementary Fig. S3e,f). While both cdc25A and cdc25B have been reported to bind to 14-3-3 proteins, neither forms a complex with 14-3-3γ and have been shown to bind other 14-3-3 isoforms^54^,^55^. It is possible that all of the cdc25 isoforms can regulate the increase in centrosome duplication, indeed it has been reported that all of them are required for the complete activation of cdk1/cyclinB^81^ and that multiple isoforms need to be inactivated to generate a cell cycle arrest^82^. These are also consistent with our results that over-expression of cdk1 or an active form of cdk1 could lead to an increase in centrosome duplication, as the increased activity of the cdc25 family members would remove the inhibitory phosphates from the over-expressed cdk1. However, the increase in centrosome number upon loss of 14-3-3γ is dependent on the expression of cyclin B, as suggested by the data in Fig. 2, suggesting that the effects we observe are dependent on cdk1.

Our work also demonstrates that in addition to regulating cdc25C function, 14-3-3γ also binds to other centrosomal or centrosome associate proteins that regulate centrosome function. It is also possible that the interaction of 14-3-3γ with centrosomal proteins prevents their phosphorylation by cdks, thus preventing centrosome amplification (Fig. 7a). These results suggest the probable existence of other mechanisms by which 14-3-3γ regulates centrosome duplication and biogenesis by regulating PLK4/Sas-6 mediated centriole cartwheel formation^83^,^84^, or regulating the assembly of pericentriolar matrix^85^,^86^, or the formation of γ-Tubulin-ring complex to regulate microtubule nucleation^19^. Further work is required to clarify the mechanisms by which 14-3-3 proteins regulate centrosome biogenesis, as 14-3-3 proteins are broad-spectrum phospho-Ser/Thr-binding adaptor proteins that act as signal integration nodes for multiple biochemical pathways^29^.

Multipolar cell divisions are rare and multipolar spindles are often unstable short-lived intermediates. Tumor cells have evolved mechanisms to induce centrosome clustering leading to the formation of a pseudo-bipolar spindle during mitosis. Pseudo-bipolar mitoses result in the formation merotelic chromosome attachments leading to a favorable aneuploidy and acquisition of the neoplastic phenotype^61^. However, a hypothesis that is being currently promulgated in the literature suggests that promoting excessive genetic instability could be a potential target for anti-tumor therapeutics^61^,^87^, as tumor cells are programmed to be genetically unstable due to the inactivation of checkpoint pathways [reviewed in^88^,^89^,^90^]. Thus, exploiting the addiction to genetic instability could result in increased cell death even in tumors that are normally resistant to several cytotoxic agents used in cancer therapy. Our work demonstrates that rather than inhibiting cdk1 activity, a novel way of inhibiting tumor growth might be the premature activation of cdk1 in interphase cells. This is in contrast to the paradigm generally accepted in the literature where several studies have attempted to use inhibitors of the cell-cycle kinases to inhibit tumor growth, an approach that has not achieved significant success. While our observation is based on data generated in a xenograft mouse model that lacks an intact immune system, similar experiments could not be performed in mouse genetic models as mouse cdc25C lacks the 14-3-3 binding site and does not form a complex with 14-3-3 proteins (our unpublished data). However, given the data suggesting that over-expression of B cyclins in the mouse results in tumor progression^26^ and the number of papers suggesting that cyclin B is over-expressed in human tumors as compared to normal tissue^91^,^92^,^93^,^94^,^95^,^96^, we believe that our data suggesting that the premature activation of cdk1 is a potent way of killing tumor cells, certainly has broad significance for the field of tumor therapeutics (Fig. 7b).

Overall, this study provides a conceptual framework to understand the role of 14-3-3 proteins in centrosome duplication by regulating the activity of cdc25C, cdk1 and NPM1. Our work also indicates the future possibility of development of therapeutic methods to reduce tumor growth by targeting the disruption of 14-3-3-cdc25C complex in the interphase cells, without affecting normal cells. The novel molecular basis of 14-3-3-mediated centrosome duplication and harnessing this concept to inhibit centrosome-clustering and subsequent tumor reduction opens the door towards understanding the regulation of centrosome biogenesis by myriad roles of 14-3-3 proteins, and creating novel avenues for preventing tumor growth by centrosome de-clustering.

## Materials and Methods

### Ethics Statement

Maintenance of the animal facility is as per the national guidelines provided by the Committee for the Purpose of Control and Supervision of the Experiments on Animals (CPCSEA), Ministry of Environment and Forest, Government of India. All the experiments in this manuscript have been carried out according to the approved guidelines. The animals were housed in a controlled environment with the temperature and relative humidity being maintained at 23 ± 2 °C and 40–70% respectively and a day night cycle of 12 hrs each (7:00 to 19:00 light; 19:00 to 7:00 dark). The animals were received an autoclaved balanced diet prepared in-house as per the standard formula and sterile water *ad libitum*. Mice were housed in the Individually Ventilated Cage (IVC) system (M/S Citizen, India) provided with autoclaved corn cob bedding material (Natgrit 406) procured from Natural Organics, Satara, MS, India. Protocols for the experiments were approved by the Institutional Animal Ethics Committee (IAEC) of the Advanced Centre for Treatment Research and Education in Cancer (ACTREC). The animal study proposal number is 11/2008 dated August 19, 2008.

### Cell culture and transfections

HCT116, U-2OS and HEK293 cells and the HCT116 derived vector control and 14-3-3γ-knockdown cells were cultured as described^31^. Cells were transfected with lipofectamine-LTX (Invitrogen) according to the manufacturer’s instructions. HCT116 cells transfected with the pTRIPZ constructs expressing either WT cdc25C or S216A were maintained in media containing 1 μg/ml of puromycin. Expression of cdc25C was induced by adding doxycycline to the medium at a concentration of 2 μg/ml. To perform the rescue experiments GFP14-3-3γ-R (shRNA resistant 14-3-3γ cDNA) was transfected into 14-3-3γ-knockdown cells and subsequently the cells expressing GFP construct were sorted using flow cytometry as described^97^. Centrosome number was determined as described below.

### Estimation of centrosome and spindle pole number, and determination of centrosome clustering and multi-polarity

To enrich cells in mitosis, the 14-3-3γ-knockdown and vector control cells were synchronized using 400 μM mimosine for 20 hours as described^98^, released and fixed with 4% para-formaldehyde after 12–14 hours, to allow them to enter mitosis. To determine the percentage of cells containing more than two centrosomes or spindle poles, centrosomes or spindle poles of 100 mitotic cells were counted from three independent experiments. As over-expression of cdc25C or cdc25C-S216A in 14-3-3γ-knockdown cell causes death in culture gradually after 48 hours of expression, centrosome counts were performed immediately after 48 hours of transfection. The spindle poles from 100(x3) mitotic cells were counted in 3 independent experiments to determine the number of cells with pseudo-bipolar, multi-polar or truly bipolar spindles.

### Immunofluorescence, FRET analysis and confocal microscopy

To determine the localization of proteins, different cell types were grown on glass cover slips. Cells were transfected with combinations of fluorescence proteins or labeled with fluorophore-conjugated antibodies. Cells were fixed with 4% para-formaldehyde and permiabilized with 0.3% tritonX-100. 0.05% DAPI was used to stain nuclei. Argon, Helium/Neon and diode lasers were used to capture images on a Carl Zeiss LSM 510 Meta confocal microscope. Images were captured under the oil immersion objectives of LSM510 meta (Carl Zeiss) confocal microscope, at 630X or 1000X magnification with 2X to 4X digital zoom. All the images were captured after background nullification with secondary antibodies. Images were processed using the LSM510 software. FRET measurements were performed using the sensitized emission method in fixed cells using samples: Donor only (GFP or Alexa-Fluor-488), Acceptor only (dsRed or Alexa-Fluor-546) and FRET sample. Following images were acquired for FRET corrections and efficiency calculations: (1) Acceptor Only using Acceptor filter set. (2) Acceptor Only using FRET filter set. (3) Donor Only using Donor filter set. (4) Donor Only using FRET filter set. (5) FRET Specimen Only using FRET filter set. All the images were captured at X630 magnification in 12-bit format using Zeiss LSM 510 Meta confocal laser scanning microscope. The images were acquired using following lasers: Donor excitation using 488 nm Argon laser line while acceptor excitation using 543 nm Helium Neon laser line. Images acquired were further processed using LSM 510 image examiner software. The nomenclature and equations for FRET calculations are as previously described^43^ and the FRET protocol was obtained from the Centre for Optical Instrumentation laboratory, Wellcome Trust Centre, University of Edinburg^99^. FRET Corrections: (i) Acceptor in FRET channel (Co-efficient A) = Average intensity of Acceptor only using FRET set/Average intensity of Acceptor only using acceptor set. (ii) Donor in FRET channel (Co-efficient B) = Average intensity of Donor only using FRET filter set/Average intensity of Donor only using Donor filter set. (iii) Average FRET efficiency = FRET Specimen − (A * FRET Specimen using Acceptor filter set) − (B * FRET Specimen using Donor filter set) * 100.

### Electron microscopy

To study centrosome amplification and organization in higher magnification 14-3-3γ-knockdown and vector-control cells were visualized under transmission electron microscope. Synchronized cells in S-phase were fixed with 3% glutaraldehyde, washed with 0.1 M of sodium cacodylate and post fixed with 1% osmium tetra oxide (Tedpella). Cultures were dehydrated and processed. Grids were contrasted with alcoholic uranyl acetate for 1 minute and lead citrate for half a minute. The grids were observed under a Carl Zeiss LIBRA120 EFTEM transmission electron microscope, at an accelerating voltage of 120KV and at 25000X magnification. Images were captured using a Slow Scan CCD camera (TRS, Germany).

### Preparation of metaphase plates

Cells were arrested in mitosis by growing them in Colcemid (0.1 μg/ml) for 2 hours and were incubated in a hypotonic solution (0.075 M KCl) for 15–25 minutes at 37 °C. Metaphase spreads were generated by dropping the cells from a height on frosted glass slides and chromosomes were stained with Giemsa and imaged under 100X objective of the AxioImager Z1 upright microscope (Carl Zeiss).

### Soft Agar Assays

Soft agar assays for the 14-3-3γ-knockdown and vector-control cells were performed as previously described^100^. To determine whether cdc25C over-expression led to a decrease in transformation, the 14-3-3γ-knockdown cells were transfected with doxycycline inducible constructs for EGFP, EGFP-cdc25C and EGFP-S216A. Transfected cells were selected in 0.5 μg/ml puromycin. 72 hours post selection, the cells were harvested by trypsinization and 10,000 cells plated in soft agar containing puromycin at a concentration of 0.5 μg/ml in the presence or absence of 2 μg/ml doxycycline in triplicate. The remaining cells were cultured in regular media containing puromycin at a concentration of 0.5 μg/ml in the presence or absence of 2 μg/ml doxycycline. The cells were harvested and protein extracts were prepared as described^27^ and resolved on SDS-PAGE gels for Western blot analysis with antibodies to GFP.

### Tumour formation in immunocompromised mice

For the present study, we used NOD.CB17-*Prkdc*^*scid*^/NCrCrl (NOD-SCID mice) or BALB/c Nude mice (CAnN.Cg-*Foxn1nu*/Crl). The foundation stock of the immuno-compromised mice was procured from Charles River Laboratories, Willington, USA. All animal studies were approved by the Institutional Animal Ethics committee (IAEC) constituted under the guidelines of the CPCSEA, Government of India. 10^6^ HCT116 derived 14-3-3γ-knockdown and vector-control cells were re-suspended in DMEM medium without serum and injected subcutaneously in the dorsal flank of 6–8 weeks old NOD-SCID mice (obtained from ACTREC animal house facility). Five mice were injected for each clone. Tumor formation was monitored at intervals of 2–3 days and tumor size was measured by Vernier calipers. Tumor volume (mm^3^) was calculated by the formula ½ LV^2^ where L is the largest dimension and V its perpendicular dimension, as previously reported^100^. For the tumor reversal experiment, nude mice were injected with 10^6^ cells and tumor volumes measure as mentioned above. One set of mice were given 2 mg/ml dox + 5% sucrose in drinking water (protected from light). The water was changed every 3 days.

### Plasmids and constructs

The shRNA constructs targeting 14-3-3ε and 14-3-3γ and the shRNA resistant 14-3-3γ cDNA were described previously^31^,^101^. Published shRNA sequences for cdc25C^102^, cdc25B^103^, cdc25A^104^, cdk1^105^ and cdk2^106^ (Table 1) were cloned in pTU6IIA^100^ digested with AgeI and XhoI (New England Biolabs). The 5′ and 3′ oligonucleotides were annealed and phosphorylated at both the ends using T4 polynucleotide kinase (Fermentas). Oligos were designed in such a way that AgeI and XhoI restriction sites remained at the two termini. The annealed oligos were cloned into the pTU6 vector digested with AgeI and XhoI The shRNA cassettes was excised with EcoRI and XhoI and cloned into pEGFP-f (Clontech). The GFP-centrin construct^107^, the cdk1 expression constructs^108^ and the CFP-lamin construct^109^ have been described previously. DsRed-14-3-3γ was generated by removing stop-codon from 14-3-3γ cDNA by PCR and cloned between Nhe1 and BamH1 of the pDsRedN1 vector (Clontech). Cdc25C and cdc25C-S216A mutant were cloned into pEGFPN1 (Clontech) and subsequently sub-cloned as EGFP fusions into pTRIPZ (Open Biosystems). WT NPM1 was cloned into the HindIII/XbaI sites of the pFLAG-CMV2 vector and the T199A and T199D mutants were generated by site-directed mutagenesis (Stratagene). Reverse transcriptase coupled polymerase chain reactions (RT-PCR) for the different 14-3-3 genes or GAPDH as a loading control were performed as described^100^.

### Antibodies

Primary antibodies for 14-3-3γ (CG31; Abcam ab76525; dilution 1:2500), 14-3-3ε (T16; Santacruz sc1020; dilution 1:1000), 14-3-3σ (CS112 tissue culture supernatant 1:50), β-actin (Sigma A5316; dilution 1:5000), GFP (Clontech 632375; dilution 1:15,000), NPM1 (Invitrogen 325200; dilution 1:5000), phospho-T199 NPM1 (Abcam ab81551; dilution 1:2000), Aurora A (Invitrogen 458900, dilution 1:1000), p-T288 Aurora A (Cell signaling technology 3079, dilution 1:1000) and h-Sas6 (110 dilution 1:3000) were used for Western blot experiments. The secondary goat anti-mouse HRP (Pierce) and goat anti-rabbit HRP (Pierce) antibodies were used at a dilution of 1:2500 for Western blot analysis. Primary antibodies for Cep-170 (Invitrogen 41-3200; dilution 1:50), γ-Tubulin (Sigma T3559; dilution 1:200), α-tubulin (Abcam ab7291; dilution 1:500), centrin1 (Abcam ab11257; dilution 1:50), Ninein (Abcam ab4447; dilution 1:50), 14-3-3γ (CG31; Abcam ab76525; dilution 1:200) antibodies were used for immunofluorescence. Secondary antibodies (conjugated with Alexafluor-568, Alexafluor-546, Alexafluor-455 from Molecular probes, Invitrogen; dilution 1:100) were used for immunofluorescence studies.

### Cell cycle analysis

To determine the time point of centrosome duplication, 14-3-3γ-knockdown and vector-control cells were arrested at G1/S boundary and released at different intervals afterwards. Cells were synchronized at G1/S phase by 400 μMM mimosine for 20 hours^98^. Cells were washed twice with PBS and then fed with complete medium. Cells were harvested by trypsinization at 0, 2, 4, 6, 8 and 10 hours post release, fixed with 100% ethanol and stained with propidium iodide (Sigma) and the cell cycle profiles were acquired on a FACS Calibur (BD Biosciences) and analyzed using MODFIT software^31^. Protein extracts prepared from another aliquot of cells were used to determine the levels of CyclinB1, 14-3-3γ and actin by Western blot analysis. At each time point, cells were stained with antibodies to centrosome proteins and centrosome number determined as described above.

### MALDI-TOF/TOF mass spectrometry

Lysates 14-3-3γ-knockdown and vector-control cells were used in GST-pulldown using GST-14-3-3γ (pGEX-3X, GE) as bait. Pulldown fractions were resolved in 6–12% gradient SDS-PAGE and gels were visualized by colloidal coomassie stain (PAGE blue, Fermentas). Bands of differential intensities were excised and treated with 30 mM potassium ferricyanide and 100 mM sodium thiosulfate solution. The gel pieces were reduced with 10 mM DTT. Rehydrated and reduced gel pieces were trypsinized in 20 μg/ml Trypsin (proteomics grade, Sigma, 5266) in 25 mM ammonium bicarbonate at 37 °C overnight. Extraction of the in-gel digested peptides was performed with 5% v/v trifluoro acetic acid in 50% v/v acetonitrile. 1 μl of recovered peptides and 1 μl of peptide matrix solution (20 mg/ml HCCA in 0.1% v/v TFA in 50% v/v acetonitrile) were spotted onto sample target plate. External calibration was prepared by mixing peptide standard mixture and peptide matrix solution similarly. Mass spectra were acquired by MALDI-TOF/TOF mass spectrometer (Bruker Daltonics, Ultraflex II) on reflector ion positive mode. MASCOT database search engine (version 2.2.03) was used for comparing peptide masses with those in NCBInr protein database (database version: NCBInr_20080812.fasta) in *Homo sapiens*. Searches were carried out with trypsin digestion, one missed cleavage, fixed carbamidomethylation of cysteine residues and optional oxidation of methionine with 100-ppm mass tolerance for mono-isotopic peptide masses.

### *In vitro* kinase assays

The cdk1/cyclinB1 enzyme was purchased from ProQinase. 1 μg of bacterially expressed recombinant WT NPM1-his_6_ or T199A-NPM1-his_6_ was incubated along with about 4 ng of cdk1/cyclinB1 enzyme in a 20 μl reaction mixture containing 50 mM Tris-HCl, 100 mM NaCl, 0.1 mM EGTA, 10 mM MgCl_2_, 0.2% β-mercaptoethanol, and [γ-^32^P] ATP. The reaction mixture was resolved on a 12% SDS-PAGE and autoradiography was performed.

### MTT assays

To determine the viability of vector control and 14-3-3γ-knockdown cells expressing cdc25C or cdc25C-S216A, the colorimetric MTT metabolic activity assay was performed. The control and knockdown cells were transfected with doxycycline inducible (and Puromycin resistant) EGFP, EGFP-cdc25C WT and EGFP-cdc25C-S216A constructs. 24 hours post transfection, cells were washed with PBS and fed with fresh media containing selection antibiotic (DMEM + Puromycin). 60 hours post selection, 2000 cells of each lines were seeded in 96-well microtiter plate. After the cells had adhered (~24 hours), media was changed to DMEM + Puromycin + Doxycycline, in order to induce the expression of cdc25C. The day of addition of doxycycline was considered as day 0 and the MTT assay was performed across 6 days. For the MTT assay, 20 μL of 5 μg/mL MTT [3-(4,5-Dimethylthiazol-2-yl)-2,5-Diphenyltetrazolium Bromide)] reagent was added to each well. 4 hours post addition of MTT, 100 μL of 10% SDS in HCl was added to the wells and incubated overnight. Absorbance of each well was measured at 540 nm/690 nm to assess viability. Percentage of cell viability is depicted as relative to that of day 0.

## Additional Information

**How to cite this article**: Mukhopadhyay, A. *et al*. 14-3-3γ Prevents Centrosome Amplification and Neoplastic Progression. *Sci. Rep.* **6**, 26580; doi: 10.1038/srep26580 (2016).

## Supplementary Material

Supplementary Information

## Figures and Tables

**Table 1:** Sequences of oligonucleotide primers.

**Oligonucleotide**	**Sequence**
cdk1a	CCGGATGGGGATTCAGAAATTGATCAGTTCTCGATCAATTTCTGAATCCCCATTTTTTTC
cdk1b	TCGAGAAAAAAATGGGGATTCAGAAATTGATCGAGAACTGATCAATTTCTGAATCCCCAT
cdk2a	CCGGAGCTGTGGACATCTGGAGCCTAGTTCTCAGGCTCCAGATGTCCACAGCTTTTTTTC
cdk2b	TCGAGAAAAAAAGCTGTGGACATCTGGAGCCTGAGAACTAGGCTCCAGATGTCCACAGCT
14-3-3β Fwd	GGTATCTTTCTGAAGTGGC
14-3-3β Rev	GCTACAGGCCTTTTC
14-3-3γFwd	GAGCCACTGTCGAATG
14-3-3γ Rev	CGCTGCAATTCTTGATC
14-3-3σ Fwd	GCAGCCTTCATGAAAG
14-3-3σ Rev	CCCTTCATCTTCAGGTAG
14-3-3ζ Fwd	GTTCTTGATCCCCAATGC
14-3-3ζ Rev	CTCTGGGGAGTTCAGAATC
GAPDH Fwd	TGCATCCTGCACCACCAACT
GAPDH Rev	CGCCTGCTTCACCACCTTC
Cdc25C-1a (shRNA)	CCGGTGAAGAGAATAATCATCGTGTTTTCAAGAGAAACACGATGATTATTCTCTTCTTTTTC
Cdc25C-1b (shRNA)	TCGAGAAAAAGAAGAGAATAATCATCGTGTTTCTCTTGAAAACACGATGATTATTCTCTTCA
Cdc25B-1a (shRNA)	CCGGTAATCCTCCCTGTCGTCTGAATTTCAAGAGAATTCAGACGACAGGGAGGATTTTTTTC
Cdc25B-1b (shRNA)	TCGAGAAAAAAATCCTCCCTGTCGTCTGAATTCTCTTGAAATTCAGACGACAGGGAGGATTA
Cdc25A-1a (shRNA)	CCGGTAGCAACCACTGGAGGTGAAGTTCAAGAGACTTCACCTCCAGTGGTTGCTTTTTTC
Cdc25A-1b (shRNA)	TCGAGAAAAAAGCAACCACTGGAGGTGAAGTCTCTTGAACTTCACCTCCAGTGGTTGCTA

Sequences of oligonucleotide primers used for designing shRNA constructs and performing RT-PCR assays.
