# Supplementary material for: 14-3-3γ Prevents Centrosome Amplification and Neoplastic Progression
Source: Sci Rep. 2016 Jun 2;6:26580. doi: 10.1038/srep26580 (PMC4890593; doi:10.1038/srep26580)
Supplement: Supplementary Information [file srep26580-s1.pdf]

Supplementary Figures.

14-3-3γ Prevents Centrosome Amplification and Neoplastic Progression.

Amitabha Mukhopadhyay[1, ¶], Lalit Sehgal[1, §, ⌘], Arunabha Bose[1, §], Anushree Gulvady[1,¥], Parijat Senapati[4], Rahul Thorat[1], Srikanta Basu[1], Khyati Bhatt[1], Amol S. Hosing[1], Renu Balyan[2], Lalit Borde[3], Tapas K. Kundu[4] and Sorab N. Dalal[1, *].

[1] Advanced Centre for Treatment Research and Education in Cancer, Tata Memorial Center, Mumbai 410210, India.
[2] National Institute of Immunology, New Delhi 110067, India.
[3] Department of Biological Sciences, Tata Institute of Fundamental Research, Mumbai 400005, India.
[4] Transcription and Disease Laboratory, Molecular Biology and Genetics Unit, Jawaharlal Nehru Centre for Advanced Scientific Research, Bangalore 560064, India.

*** Corresponding author**
Sorab N. Dalal, KS215, Advanced Centre for Treatment Research and Education in Cancer, Tata Memorial Center, Mumbai 410210, India. Email. sdalal@actrec.gov.in
[§] These authors contributed equally to the work.

Current address
[¶] Institute of Biophysical Dynamics, University of Chicago, Chicago, IL 60637, USA.
[⌘] Department of Lymphoma and Myeloma, MD Anderson Cancer Center, Texas, TX 77030, USA.
[¥] Department of Cell and Developmental Biology, State University of New York Upstate Medical University, Syracuse NY 13210, USA.

Mukhopadhyay et.al., Supplementary Figure 1

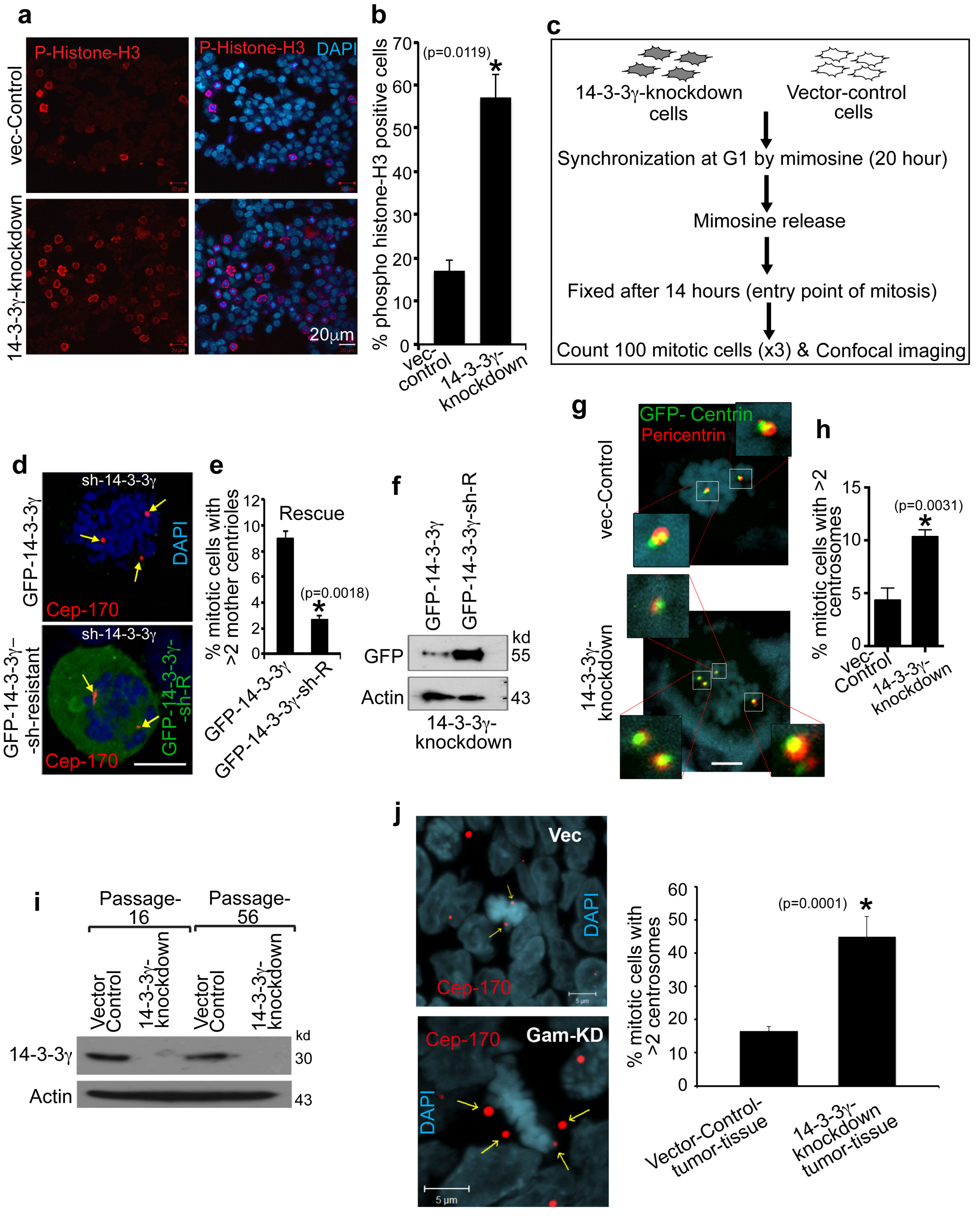

Supplementary figure S1. Depletion of 14-3-3γ results in higher mitotic index and centrosome amplification. (**a-b**) The 14-3-3γ-knockdown and vector-control cells were synchronized with mimosine and released into the cell cycle. 12 hours post release from mimosine they were stained with antibodies to phospho-Histone-H3 (pH3) (red) and counter stained with DAPI (blue). The number of pH3 positive cells was determined in three independent experiments and the mean and standard deviation plotted. Student's t test (2 sample unequal variance) was used to determine p-value; $p<0.05$ (*). (**c**) Experimental outline for determining centrosome number in mitotic cells. (**d-f**) 14-3-3γ knockdown cells were transfected with shRNA-resistant-GFP-14-3-3γ or GFP-14-3-3γ. GFP expressing cells were sorted and stained with antibodies to Cep-170, co-stained with DAPI and centrosome number was determined. Representative images are shown (**d**) and the mean and standard deviation from three independent experiments is plotted (**e**). Protein extracts, prepared from the sorted cells, were resolved by SDS-PAGE followed by immuno-blotting with the indicated antibodies (**f**). (**g-h**) Vector control or 14-3-3γ knockdown cells were transfected with GFP-Centrin and stained with antibodies to Pericentrin, co-stained with DAPI followed by confocal microscopy and centrosome number was determined. Representative images are shown (**g**) and the mean and standard deviation from three independent experiments is plotted (**h**). Images were captured at 630x magnification and 2x digital zoom under confocal microscope. Significance was derived using a Student's t-test (2 sample unequal variance) with $p< 0.05$. Scale bar indicate 10μm, unless mentioned. (**i**) Cell lysates of vector-control and 14-3-3γ-knockdown cells from passage-16 and passage-56 were resolved by SDS-PAGE and immuno-blotted with antibodies to 14-3-3γ. The levels of 14-3-3γ were reduced in 14-3-3γ-knockdown cells at passage-16 and passage-56. Actin served as a loading control. **(j)** Tumor tissue from tumors generated with the vector control and 14-3-3γ-knockdown cells were stained with antiboides to Cep-170, counter-stained with DAPI and centrosome number determined as described. The mean and standard deviation are plotted. p values were obtained using a student's t test.

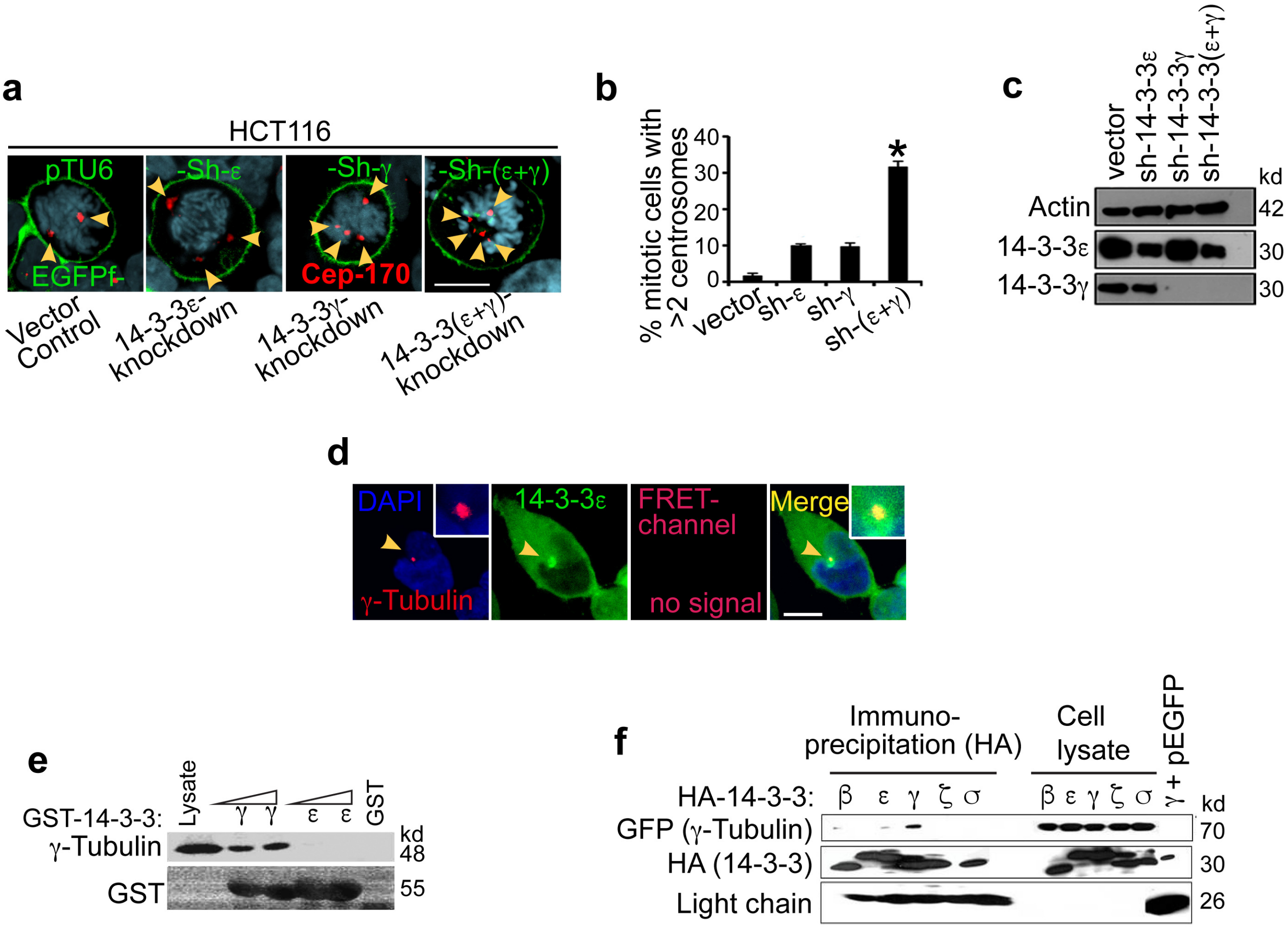

g

Protein Identified	Accession number	Molecular weight(kd)	Total Peaks	Matched Peaks	Sequence covered (%)	Score	p-value
**GCP-2**	AF042379	102.53	33	7	20	55	0.0489
**KIF5B**	P33176	110.35	59	24	29	142	1.3e-10
**KLC-2**	Q9H0B6	71.08	52	6	15	56	0.047
**IRS1**	P35568	132.7	28	13	18	98	3.5e-06
**Cbl-c**	P22681	100.89	40	11	14	63	0.01
**HSC71**	P11142	71	59	9	18	56	0.05
**ZNF224**	Q9NZL3	84.87	35	10	18	68	0.0034

Other information:

Enzyme used : Trypsin; **Database version:** Swiss Prot_56.0 fasta;
Search engine (version): Mascot (2.2.03);
Conditions for search: Global modifications - Carbamidomethylation;
Variable modifications - oxidation of methyl groups; **Tolerance (Dalton):** 1; **PPM:** 100;
Mass cleavage: 1; **Species:** *Homo sapiens.*

Supplementary figure S2. Centrosome amplification is increased due to dual knockdown of 14-3-3γ and 14-3-3ε. (**a**) HCT116 cells were transfected with either EGFPf-sh-14-3-3γ or EGFPf-sh-14-3-3ε or with a combination of both. “sh” represents “short hair-pin”, indicating the expression of vector-driven sh-RNA from the plasmid constructs. EGFPf-sh-pTU6 was used as control. Transfected cells expressing shRNA were identified by EGFPf (farnesylated) expression on the cell membrane. Centrosomes were stained with anti-Cep-170 antibody and nuclei by DAPI. Images were captured at 630x magnification and 2x digital zoom under confocal microscope. Scale bar represents 10μm. (**b**) Percentage of cells containing >2 centrosomes were counted from 100 cells and 3 independent experiments. Standard error of means are plotted on the bar diagram and significance was derived from Student’s t-test (2 sample unequal variance) with $p < 0.05$. (**c**) Reduction in 14-3-3γ or 14-3-3ε by shRNA is shown in the Western blot image. (**d**) 14-3-3ε and γ-Tubulin in HCT116 cells were stained with the specific primary antibodies. Nuclei were co-stained with DAPI. Sensitized emission FRET analysis was performed with secondary antibodies, conjugated with FRET-pair Alexa-Fluor-488 (donor) and Alexa-Fluor-546 (acceptor) fluorophore. Scale bar represents 10μm. (**e**) Bacterially expressed, GST-fused 14-3-3γ or 14-3-3ε in increasing concentrations, were incubated with protein extracts prepared from HCT116 cells and the reactions resolved on SDS-PAGE gels followed by Western blots with antibodies to γ-Tubulin. 5% of the protein extract was loaded on the gel and GST served as a negative control. (**f**) HCT116 cells were co-transfected with GFP-γ-Tubulin and HA-14-3-3 isoform. Immunoprecipitation was performed with anti-HA antibody and immuno-blotting with anti-GFP antibody. EGFP was used as negative control. Expression of HA-14-3-3 isoforms were identified by anti-HA antibody; amount of antibodies used for the IP, is shown in the antibody light-chain panel. (**g**) GST14-3-3γ-bound protein fractions from 14-3-3γ-knockdown and vector-control cells were resolved by SDS-PAGE, stained with colloidal coomassie stain. Bands with differential intensities were processed for peptide mass fingerprinting. The table describes the details of the parameters used to identify proteins by MALDI-TOF mass spectrometry. All Western blots were carried out under same experimental conditions and the full-length blots are shown in Supplementary figure S6.

Mukhopadhyay et.al., Supplementary Figure 3

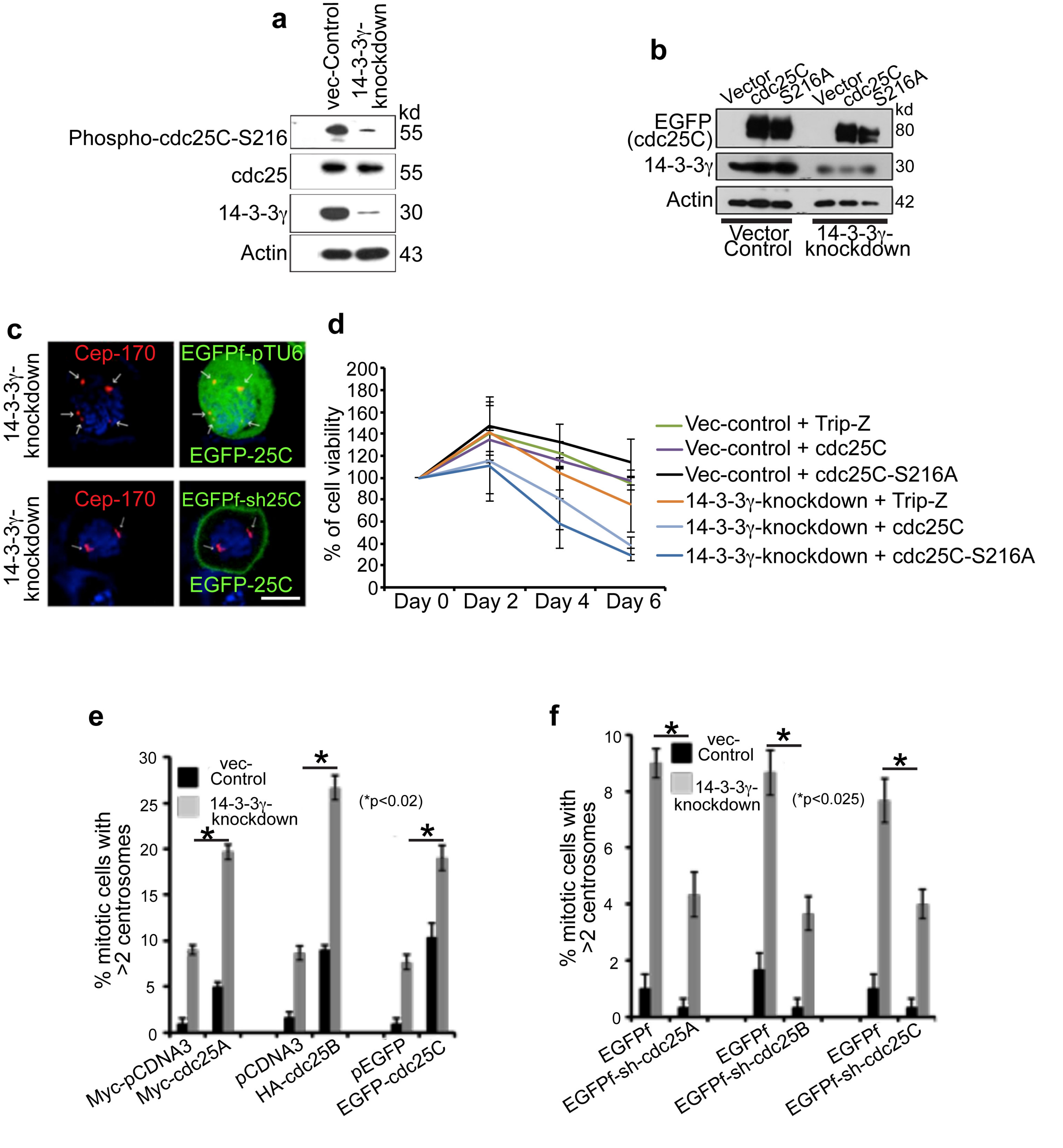

Supplementary figure S3. Expression of active cdc25C-S216A (14-3-3-binding deficient) results in an increased centrosome amplification and cell death. (**a**) Protein extracts prepared from the vector control or 14-3-3γ-knockdown cells were resolved on SDS-PAGE gels followed by Western blotting with the indicated antibodies. Note the decrease in phosphorylation of the S216 residue in the 14-3-3γ knockdown cells. Western blots for actin serve as a loading control. (**b**) The 14-3-3γ knockdown cells or the vector controls were transfected with the indicated constructs. Post-transfection, protein extracts prepared from these cells were resolved on SDS-PAGE gels followed by Western blotting with the indicated antibodies. Note that cdc25C and S216A are expressed at equivalent levels in all transfections. Western blots for actin serve as loading control. (**c**) Confocal images are showing down-regulation of cdc25C. EGFP-cdc25C was co-expressed in the cells with the knockdown construct, EGFPf-sh-cdc25C (mentioned as sh25C in the images). Centrosomes were stained with anti-Cep-170 antibody. Arrows are pointing centrosomes. Images were captured at 630x magnification and 2x digital zoom under confocal microscope. (**d**) Percentage of viability of vector-control and 14-3-3γ-knockdown cells, upon expression of cdc25C and cdc25C-S216A, was measured by MTT assay. Cdc25C and cdc25C-S216A was conditionally expressed from pTripZ vector-backbone by doxycycline induction. All experiments were performed in triplicate, and the relative cell viability was expressed as a percentage relative to the untreated control cells. (**e**) 14-3-3γ-knockdown and vector control cells were transfected with the epitope tagged constructs of cdc25A, B and C. Post transfection the cells were stained with anti-Cep-170 antibody and the percentage of cells with more than 2 centrosomes were determined from 100 mitotic cells from 3 different experiments and the mean and standard error are plotted; Student's t test (2 sample unequal variance) was used to determine p-value; $p<0.05$ (*). (**f**) Levels of cdc25A, B and C isoforms were reduced by individual vector-driven shRNA constructs. Percentage of mitotic cells containing more than 2 centrosomes was determined from the transfected cells expressing the farnesylated-EGFP at the cell boundary. All Western blots were carried out under same experimental conditions and the full-length blots are shown in Supplementary figure S6.

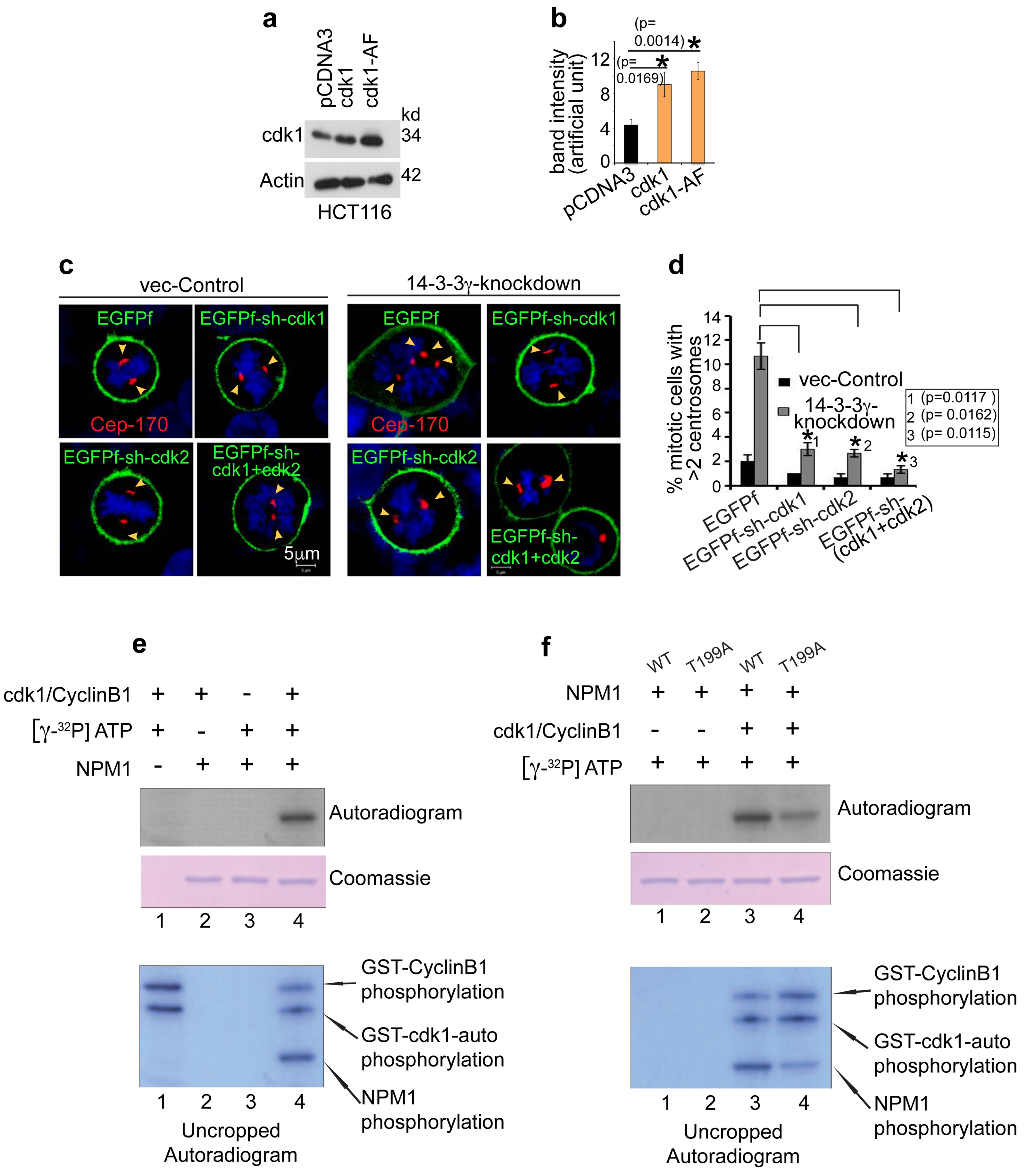
a
pCDNA3
cdk1
cdk1-AF
kd
cdk1
34
Actin
42
HCT116
b
(p= 0.0014)
(p= 0.0169)
band intensity (artificial unit)
12
8
4
0
pCDNA3
cdk1
cdk1-AF
c
vec-Control
14-3-3γ-knockdown
EGFPf
EGFPf-sh-cdk1
Cep-170
EGFPf-sh-cdk2
EGFPf-sh-cdk1+cdk2
5μm
d
% mitotic cells with >2 centrosomes
14
12
10
8
6
4
2
0
vec-Control
14-3-3γ-knockdown
1 (p=0.0117)
2 (p= 0.0162)
3 (p= 0.0115)
EGFPf
EGFPf-sh-cdk1
EGFPf-sh-cdk2
EGFPf-sh-(cdk1+cdk2)
e
cdk1/CyclinB1 + + - +
[γ-32P] ATP + - + +
NPM1 - + + +
Autoradiogram
Coomassie
1 2 3 4
GST-CyclinB1 phosphorylation
GST-cdk1-auto phosphorylation
NPM1 phosphorylation
Uncropped Autoradiogram
f
WT T199A WT T199A
NPM1 + + + +
cdk1/CyclinB1 - - + +
[γ-32P] ATP + + + +
Autoradiogram
Coomassie
1 2 3 4
GST-CyclinB1 phosphorylation
GST-cdk1-auto phosphorylation
NPM1 phosphorylation
Uncropped Autoradiogram

Supplementary figure S4. Cdk1 overexpression induces centrosome amplification, and loss of cdk1 (or cdk2) in 14-3-3γ-knockdown cells prevents centrosome amplification. (a-b) Protein extracts from the cdk1- or cdk1-AF-transfected cells were resolved on SDS PAGE gels, followed by immuno-blotting with the indicated antibodies. pCDNA3 transfected cells were used as vector control (**a**). The degree of cdk1 over-expression was determined by densitometry using Image-J software. Relative densities were plotted from 3 independent experiments (**b**). (**c-d**) The 14-3-3γ-knockdown and vector-control cells were transfected with plasmids expressing either EGFPf alone or a combination of EGFPf and shRNA targeting either cdk1 (sh-cdk1) or cdk2 (sh-cdk2) or both the proteins. Post-transfections the cells were stained with antibodies to Cep170 and counter stained with DAPI followed by confocal microscopy. Representative image panels at 630x magnification are shown. Scale bar represents 5μM (**a**). The transfected cells were identified by EGFPf expression and centrosome number was determined as described in the materials and methods. The percentage of cells with >2 centrosomes was determined in three independent experiments. The mean and standard deviation are plotted and p values were determined using Student's t test (2 sample unequal variance). p values < 0.05 are indicated by asterisk (**d**). (**e-f**) Purified cyclinB/cdk1 was incubated with recombinant WT NPM1-his_6 or T199A-NPM1-his_6 in the presence of radio-labeled ATP. The reactions were resolved on SDS-PAGE gels, stained with coomassie blue followed by autoradiography. Bacterially expressed recombinant was used for kinase assay with cdk1/cyclinB1 complex in buffer containing [γ-^{32}P] ATP. NPM1 is phosphorylated by cyclinB/cdk1 only in the presence of radiolabeled ATP (top panel **a**). The middle panel shows the coomassie gel showing equal loading of NPM1 and the bottom panel shows the un-cropped radiograph showing auto phosphorylation of cyclinB and cdk1 **(e)**. Note that cyclinB/cdk1 does not phosphorylate the T199A mutant of NPM1 **(f)**. All Western blots were carried out under same experimental conditions and the full-length blots are shown in Supplementary figure S6.

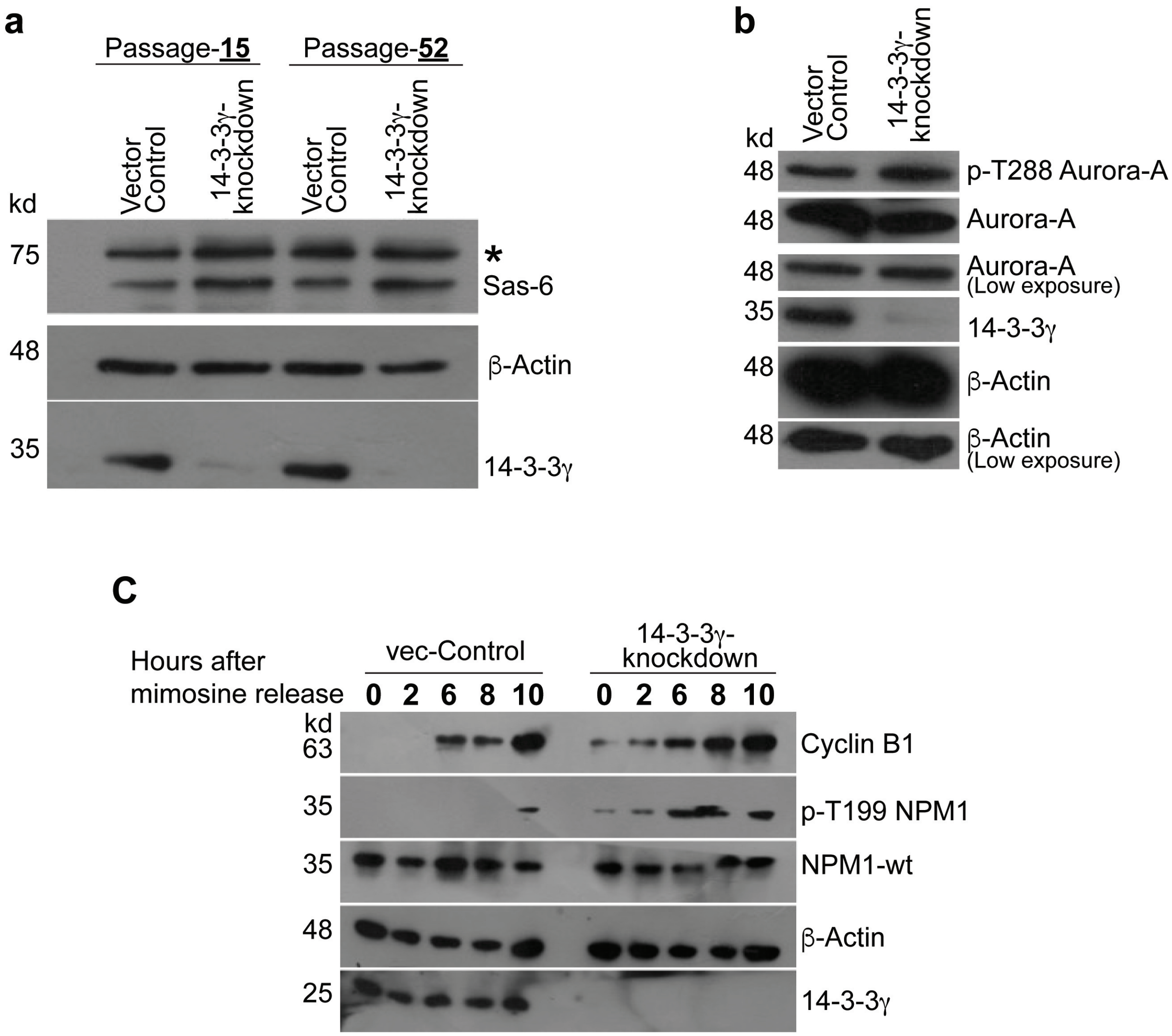

Time point (hours)	vec-control			14-3-3γ-knockdown		
	% G0/G1	% S	% G2/GM	% G0/G1	% S	% G2/GM
0	74.88	24.01	1.11	85.58	14.42	0.00
2	74.66	25.34	0.00	100.00	0.00	0.00
6	0.00	32.07	67.93	15.54	84.46	0.00
8	3.36	23.01	73.63	2.42	27.08	70.5
10	1.35	21.04	77.61	6.19	22.41	71.4

Supplementary figure S5. NPM1 phosphorylation peaks during early S-phase in the 14-3-3γ-knockdown cells. (**a**). Protein extracts from the vector control or 14-3-3γ-knockdown cells were resolved on SDS-PAGE gels followed by Western blotting with the indicated antibodies. * indicates a non-specific band identified by the Sas-6 antibody. Western blots for actin served as a loading control. (**b**). Protein extracts from the vector control or 14-3-3γ knockdown cells synchronized in mitosis by treatment with nocodazole were resolved on SDS-PAGE gels followed by Western blotting with the indicated antibodies. Note the increase in the levels of activated Aurora A (pT288) while the levels of total Aurora A remain unchanged. Western blots for actin served as a loading control. (**c**). Protein extracts prepared from synchronized cell populations of the vector control or 14-3-3γ knockdown cells were resolved on SDS-PAGE gels followed by Western blotting with the indicated antibodies. Western blots for actin served as a loading control. The percentage of cells in the different phases of the cell cycle phases is shown in the table. All Western blots were carried out under same experimental conditions and the full-length blots are shown in Supplementary figure S6.

Mukhopadhyay et.al., Supplementary Figure 6

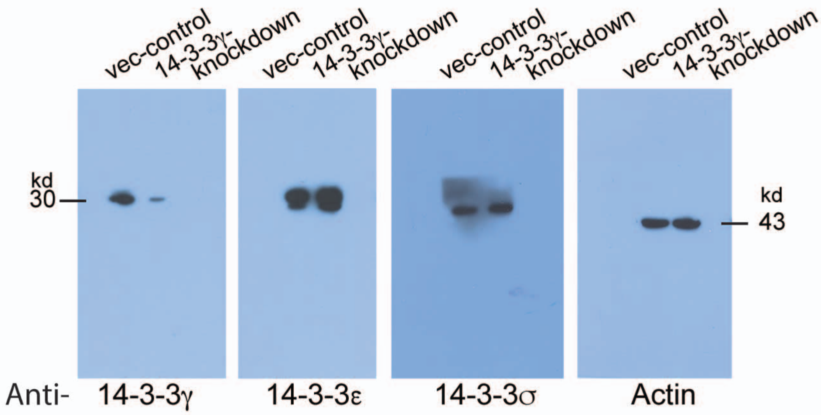

Figure 1a

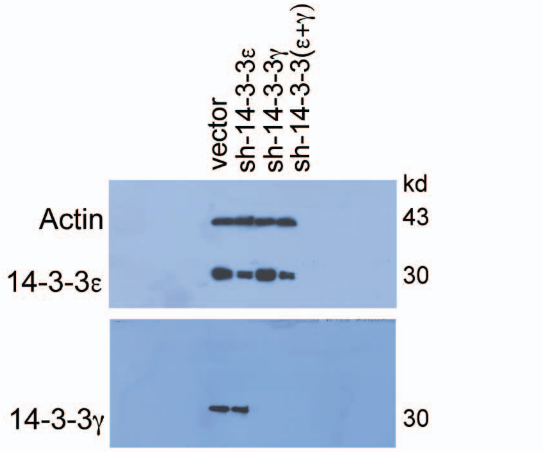

Figure Supplementary 2c

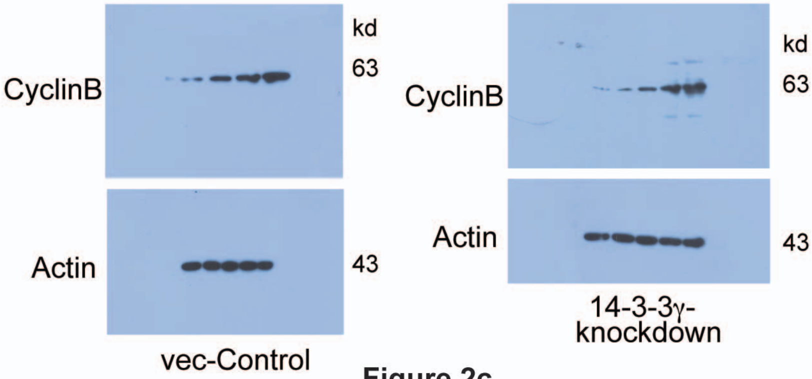

Figure 2c

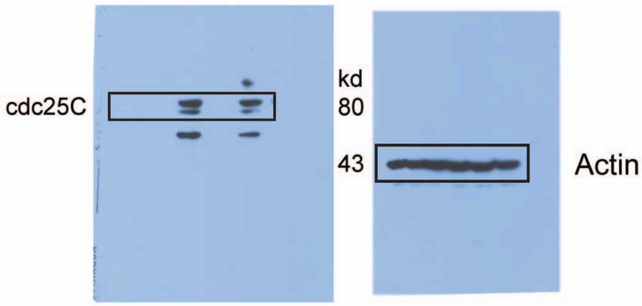

Figure 6f

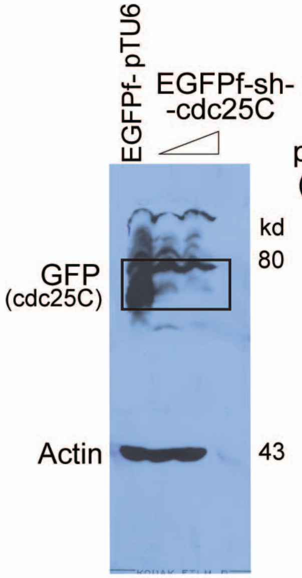

Figure 4g

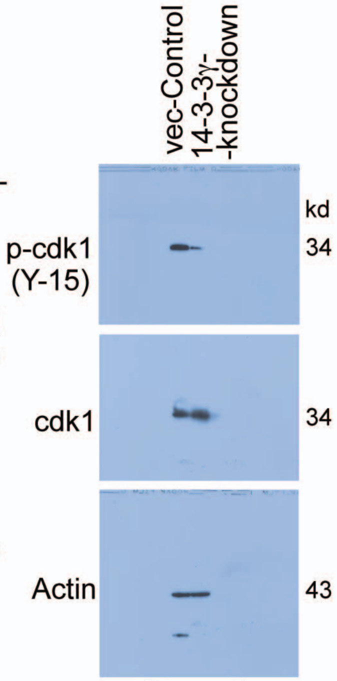

Figure 5a

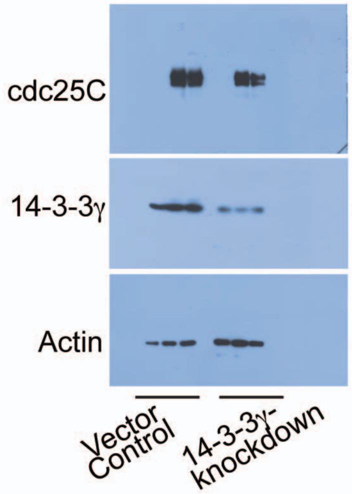

Figure Supplementary 3b

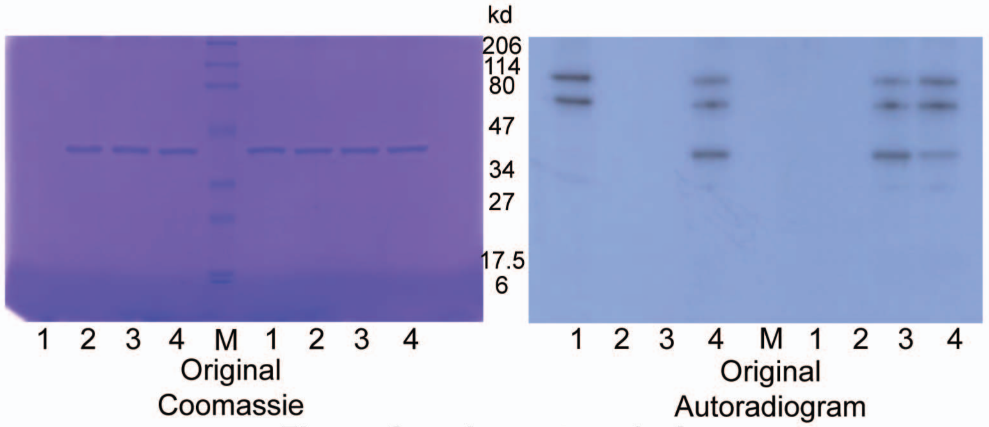

Figure Supplementary 4e-f

Supplementary figure S6. Full length blots for all the gels shown in the main manuscript. The full-length blots are shown for all the figures in the main manuscript. Western blots were performed with the indicated antibodies. The boxed region indicates the cropped area in the figure. Note that the figure number is indicated for each blot.
